# Exploring the use of internal and externalcontrols for assessing microarray technical performance

**DOI:** 10.1186/1756-0500-3-349

**Published:** 2010-12-28

**Authors:** Katrice A Lippa, David L Duewer, Marc L Salit, Laurence Game, Helen C Causton

**Affiliations:** 1 Chemical Science and Technology Laboratory.National Institute of Standards and Technology (NIST) Gaithersburg, Maryland 20899 USA; 2 MRC Clinical Sciences Centre (CSC) Imperial College Microarray Centre Hammersmith Hospital Campus London W12 0NN, UK; 3 Department of Biological Sciences Columbia University New York, NY 10027 USA

## Abstract

**Background:**

The maturing of gene expression microarray technology and interest in the use of microarray-based applications for clinical and diagnostic applications calls for quantitative measures of quality. This manuscript presents a retrospective study characterizing several approaches to assess technical performance of microarray data measured on the Affymetrix GeneChip platform, including whole-array metrics and information from a standard mixture of external spike-in and endogenous internal controls. Spike-in controls were found to carry the same information about technical performance as whole-array metrics and endogenous "housekeeping" genes. These results support the use of spike-in controls as general tools for performance assessment across time, experimenters and array batches, suggesting that they have potential for comparison of microarray data generated across species using different technologies.

**Results:**

A layered PCA modeling methodology that uses data from a number of classes of controls (spike-in hybridization, spike-in polyA+, internal RNA degradation, endogenous or "housekeeping genes") was used for the assessment of microarray data quality. The controls provide information on multiple stages of the experimental protocol (e.g., hybridization, RNA amplification). External spike-in, hybridization and RNA labeling controls provide information related to both assay and hybridization performance whereas internal endogenous controls provide quality information on the biological sample. We find that the variance of the data generated from the external and internal controls carries critical information about technical performance; the PCA dissection of this variance is consistent with whole-array quality assessment based on a number of quality assurance/quality control (QA/QC) metrics.

**Conclusions:**

These results provide support for the use of both external and internal RNA control data to assess the technical quality of microarray experiments. The observed consistency amongst the information carried by internal and external controls and whole-array quality measures offers promise for rationally-designed control standards for routine performance monitoring of multiplexed measurement platforms.

## Background

Expression profiling using DNA microarrays is increasingly being used for clinical and diagnostic applications and in support of regulatory decision-making. These applications require the technology to be robust and reliable and that the data be well characterized [1]. The quality of data generated varies considerably between laboratories [2,3] as well as between platforms [4,5]. One initiative working to provide tools for technical performance assessment of microarray gene expression data is the External RNA Control Consortium (ERCC) [6-9]. The external, "spike-in" controls from this group are intended to be informative about the quality of a gene expression assay independent of microarray platform, experiment, or species. This paper presents evidence that the spike-in controls carry the essential quality information about an experiment. Data obtained from spiked-in controls was compared with that carried by full-array quality metrics, which typically depend on platform, experiment, and species. These results support the proposition that spike-in controls can be used on their own as tools for assessing data quality and comparing data generated as part of different experiments.

Data quality can be assessed at a number of stages within the microarray experiment (from the integrity of the biological sample to the accessibility of the data stored in a databank repository) [10]. Few universal data quality metrics are available as there are a large number of array types, labeling methods, scanner types, and statistical approaches available to summarize and analyze the data. The determination of integrated whole-array data quality indicators is not yet a standard practice, and is considered an important research topic area in biostatistics [11,12], as highlighted by Brettschneider et al. [13]. The need for better quality metrics is not limited to gene expression measurements generated using microarrays: a number of other high throughput technologies (e.g., multiplex protein arrays) lack obvious simple scalar metrics that can be used to assess quality [14,15].

A number of initiatives including the Microarray Quality Control (MAQC) project of the FDA http://www.fda.gov/nctr/science/centers/toxicoinformatics/maqc/ and the ERCC are working to develop reference data sets, reference RNAs, and standard external controls intended for use in the evaluation of microarray performance [6-9]. The ERCC seeks to employ external spike-in control measurements to assess technical performance with a standard set of controls in a consistent manner using metrics that can be compared across experiments, labs, platforms, and other factors as they arise. The ERCC is developing the standard controls, analysis tools, and protocols for using these controls and tools to enable consistent assessment and monitoring of technical performance.

The MAQC project has examined the use of a diverse set of external controls for a number of platforms [16], noted that external controls have yet to be widely used for performance assessment, and made recommendations for doing so. Analysis of the control signals to assess performance was largely through quantitative characterization of the slope of the signal-concentration curve. A significant observation from this work was the identification of outlier data at one participant's site using principal component analysis (PCA) of the external controls. More recent analysis of the various spike-in controls employed in the measurements for the MAQC project demonstrated promise that the spike-in controls were informative of "outlying" arrays, and that they exhibit behavior that is independent of the sample type [17].

**Figure 1:**
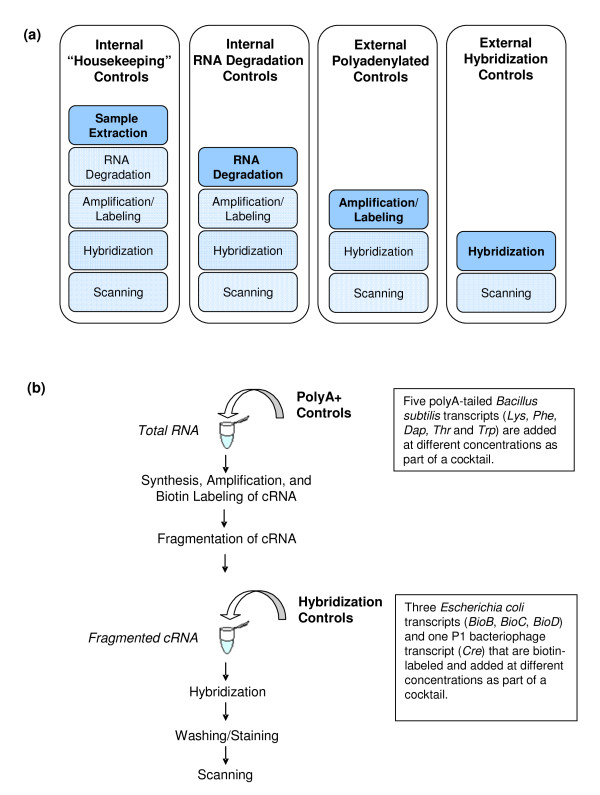
**Overview of the classes of controls (internal and external) used within a microarray experiment together with a schematic illustrating the addition of external controls at different steps during sample processing. **(a) Overview of the classes of controls (internal and external) used within a microarray experiment. Each class reports on variability originating at multiple stages. (b) Schematic protocol showing the addition of external spike-in polyA+ and hybridization controls at different steps during sample processing.

This work characterizes the internal and external control data, separate from the signal derived from the biological sample, from a microarray experiment generated on the Affymetrix GeneChip platform. The internal controls are Affymetrix-specified probesets that represent RNA degradation internal controls or "housekeeping" genes and are routinely examined to reveal the quality of the sample RNA (Figure 1a). The external, or "spike-in", controls are typically RNA transcripts produced by *in vitro *transcription that are added at a particular stage in the generation of the labeled sample transcriptome extract, at a known concentration (Figure 1a and 1b). The expression measures of these controls carry information about variation arising from a number of sources; both classes of internal controls should carry information on all of the sources of the variability in the experiment (Figure 1a). The polyA+ controls should carry information about the technical variation associated with amplification and labeling procedures only - and not variation arising from sampling - whereas the hybridization controls should carry information about variability arising from hybridization and scanning only. Employing PCA as an exploratory data analysis tool, it was anticipated that the variance structure associated with the individual steps of the microarray experiment would be revealed through the resultant scores and loadings profile of the PCA models of these four separate classes of control data.

Knowledge of the quantity of each spike added and the relative intensities of the signals can be compared against the expression measures obtained from global gene expression; this has been used as the basis of comparison between data generated on different arrays [18]. Deviations from the expected signal-concentration relationship for the spike-in controls should be informative about the technical performance of the measurement [7,19-24]. Critically, the utility of the information carried by the spike-in controls relies on the assumption that the controls act as meaningful proxies for the endogenous genes and that their behavior is representative of these genes of interest. The retrospective study undertaken here tests that assumption.

Hybridization-wise PCA was also used to compare the results of individual PCA models obtained from the control probeset data with independent laboratory measures of RNA- and hybridization-specific quality and full-array metrics [13]. Our results underscore the importance of assessing data quality and reveal some of the strengths and limitations of using spike-in and endogenous controls for assessing data quality.

## Methods

This study uses data generated on the Affymetrix GeneChip platform at the Clinical Sciences Centre/Imperial College (CSC/IC) Microarray Centre. This data is stored in, and was accessed, via the Centre's Microarray data Mining Resource (MiMiR) database [25,26]. These data were generated using a stock of external controls (polyadenylated - polyA+ controls) prepared at the Centre and distributed to individual research groups along with standard protocols for generating labeled cRNA in their own laboratories. Prelabeled hybridization controls were purchased from Affymetrix and added to the labeled samples at the Centre prior to hybridization.

The polyA+ controls are a cocktail of 5 polyA-tailed *Bacillus subtilis *transcripts (*Lys, Phe, Dap, Thr*, and *Trp*) (Figure 1b). These controls are spiked into total RNA in a fixed ratio to a fixed amount of total RNA and were carried through the sample preparation and used to monitor the efficiency of cRNA labeling and data quality. The hybridization controls (*BioB, BioC, BioD*, and *Cre *biotin-labeled transcripts) were spiked into the hybridization cocktail according to the manufacturer's instructions. They are used to align the grid and assess the efficiency of hybridization, washing and staining.

Extensive whole-array quality assurance metrics and BioConductor-based summary statistics [27-30] related to scanner/array performance and RNA quality are routinely assembled for each of the datasets with a report generated at the CSC/IC Microarray Centre. These reports are included in the MiMiR database, together with the individual hybridization files and experimental ontology and annotation information [25,26].

The Microarray Centre QA report metrics are based on .CEL file signal intensity data from GeneChip arrays and include summary statistics of all the hybridizations within a particular experiment generated using the BioConductor (BioC Release 1.9) open source software. This report provides quality assessment metrics based on: 1) Diagnostic Plots, 2) Probe-level Robust Multichip Average (RMA) Model Estimates, 3) Probe Metrics and 4) Principal Component Analysis. The first two sections include summaries of log_2 _probe RMA intensities before and after normalization as well as the RMA model fit residuals, relative log_2 _expression (RLE) and normalized unscaled standard error (NUSE) plots for the identification of outlier arrays within an experiment dataset. In addition, RNA degradation plots show the log_2 _mean intensity by probe pair position (5' end to 3' end) for each array and are used to identify samples that may have been subject to degradation. The third section, Probe Metrics, are obtained from BioConductor MAS 5.0-based statistical algorithms and are used to assess both RNA assay and hybridization performance. These include measures of scanner variability (e.g., RawQ), summarized exogenous control intensities with respect to their spike-in concentration levels, correlation measures between exogenous polyA+ controls and raw signal values, and 3'/5' ratio measures for both exogenous and endogenous controls to assess the efficiency of labeling and/or sample RNA integrity. The fourth and last section provides a simplified PCA scores plot generated from the complete set of probes (including background and all exogenous and endogenous control probes) to identify gross outliers within the experimental dataset as a whole. A recent review of these metrics as they relate to the quality assessment of microarray data after statistical processing is provided by Brettschneider et al. [13]

### Data Examined in this Study

Data from 525 hybridizations representing 22 publicly-available experiments generated over a five-year period at the CSC/IC Microarray Centre on multiple types of GeneChips were analyzed as part of this study and included human (HG-U133A, HG-U133B, HG-U133plus2), rat (RG-230_2, RAE230A, RAE230B) and mouse (MG-430_2, MOE430A, MOE430B, MG-U74v2A, MG-U74v2B, MG-U74v2C) microarrays. A single, exemplary experiment containing data from 137 Rat Genome RAE230A arrays is highlighted for this manuscript. This included data generated on different days over a 10-month period, with different experimenters, array batches, and QC measures from the whole-array QC report. This example was analyzed using PCA and the results compared to the QC and factor information available within the MiMiR database.

PCA was conducted using only data from the control-based probesets (excluding all the non-control (background) probeset signals). There are four groups, or classes, of controls, external and internal to the biological sample (exogenous and endogenous). The external controls were either polyA+ RNAs spiked into the sample before amplification and labeling or prelabeled hybridization controls spiked into the sample prior to hybridization. The internal controls are those suggested by Affymetrix as a measure of RNA degradation, and report on relatively invariant 'housekeeping' genes. Microarray probesets for the same external controls are present on all Affymetrix GeneChip arrays; probesets for the endogenous controls are organism-specific and are common to all arrays of such type (i.e., rat).

### Dataset Construction and Preprocessing

Probeset data from the individual hybridizations on RAE230A arrays (EXP_CWTA_0103_01; Array Express ID E-MIMR-222) are described in this manuscript. In brief, this experiment is a comparison of gene expression profiles of peritoneal fat of 6-week rats from 30 recombinant inbred (RI) strains derived from the spontaneously hypertensive rat (SHR/Ola) and Brown Norway congenic carrying polydactylyl-luxate syndrome (BN-Lx) strains. A single hybridization (HFB2003080611Aaa) was missing annotation for experimental QC and was thus omitted from the data analysis. A summarized version of the annotation QC information pertaining to the individual hybridizations used in this experimental dataset is provided in Additional File 1: Supplemental Table S1.

Measures representing expression were generated from the raw data using the RMA "Affy" package (Bioconductor 1.8 release) within the R environment (v 2.6.0). The data was preprocessed using background correction and quantile normalization to the global median [27]. A hybridization-specific normalization protocol was used that adjusts each probeset intensity to the 75th percentile of the non-control (background) probes and is an alternative to the quantile normalization approach typically employed with RMA-based methods. Using the expression values determined from the RMA summarization method (with only background correction), the 75th percentile of the log_2 _intensities for the background probesets associated with the individual hybridization was determined and then subtracted from the probesets of interest (i.e., hybridization and polyA+ spike-in controls and the internal Affymetrix-designated cRNA degradation and endogenous control/housekeeping gene controls). This 'brightness-scaled" normalization approach was employed to support control data aggregation across multiple array types can be generated on a similar scale can thus directly compared and permits the identification of sample-associated variability. This 75th percentile normalization was carried out for several datasets that were generated across multiple array types (data not shown) when aliquots of the same samples were hybridized to arrays of the same or different type (e.g. RAE230A and RAE230B). The 75^th ^percentile normalization was the default data analysis method for our investigations.

### Mean/SD Plots

The mean and standard deviation (SD) of the RMA values were calculated for all probesets within an experiment conducted on a single array type, comparable to other informatic methods for generating probeset-level precision metrics [2,31-33]. All mean and associated SD data pairs were employed to generate mean/SD plots that highlight control probesets associated with the hybridization, polyA+, RNA degradation, and endogenous control/'housekeeping genes' (as defined by Affymetrix for specific array types). The remaining non-control sample probesets were displayed as background for the mean/SD plots; the background average line of these data was determined as a 100-point moving average of the mean values for all the non-control probesets. All calculations were carried out using Excel code.

### Chemometric Analysis

PCA was conducted for all experimental datasets using PLS_Toolbox 4.2.1 (Eigenvector Research, Inc., Wenatchee, WA) within a MATLAB v. 7.5.0.342 (R2007b) (MathWorks, Inc., Natick, MA) computational environment. Each experimental dataset was separated into four subsets representing the: 1) spike-in hybridization controls, 2) spike-in polyA+ controls, 3) internal RNA degradation controls (Affymetrix-designated) and 4) endogenous or normalization control genes http://www.affymetrix.com/support/technical/mask_files.affx). Each PCA data subset was organized into a single data block structure with dimensions of *N*_rows _× *K*_columns _that correspond to *N *samples (hybridizations) and *K *variables (probesets) (see Table 1). Each variable in the dataset was centered to have a mean of zero but was not variance scaled. A complete list of the spike-in control probe set identifiers together with the internal RNA degradation and endogenous control probe set identifiers is provided in Additional File 1: Supplemental Table S2.

**Table 1:** Summary of the PCA models (Nsamples × Kvariables) obtained from the four control subsets of the single Rat experiment

*Data subset* *(control-type)*	*N_samples_* *(hybridizations)*	*K_variables_* *(probesets)*	*No. of PCs*	*% Variance* *Cumulative*	*RMSEC^a^*	*RMSECV^b^*
hybridization	137	18	4	94.9	0.087	0.680
polyA+	137	27	4	97.3	0.117	1.48
RNAd	137	12	3	94.6	0.143	1.05
endogenous	137	100	7	70.8	0.125	1.52

^a^Root mean square error of calibration (model)

^b^Root mean square error of cross-validation employing the venetian blinds approach

The optimal number of components to include in the PCA model was determined by the minimum of both the root mean square error of calibration (RMSEC) and of cross-validation (RMSECV) employing a venetian blinds algorithm for which the dataset were split according to their size (here 10 splits for 137 hybridizations). Datasets that contain duplicate hybridizations were subject to replicate sample trapping as the presence of related samples in test and training sets may lead to skewed cross-validation results. Here, an additional cross-validation using a random subset scheme was employed and checked for consistency with the venetian blinds approach. A summary of the PCA models including the cumulative % variance captured for each model is provided in Table 1.

## Results and Discussion

**Figure 2:**
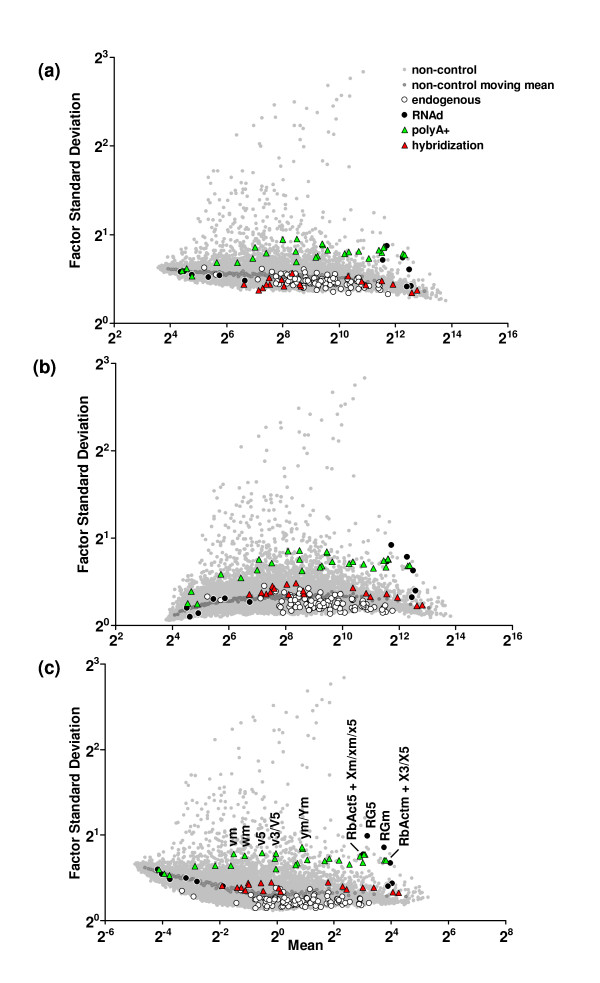
**Mean/SD plots of the RMA values for all probeset data pairs for the 137 hybridizations of the rat dataset (a) without normalization, (b) with quantile normalization and (c) with 75% percentile normalization**. The signal level scale is shifted by 2^8 ^for the 75% percentile normalization data (c). Separate symbols denote probeset data pairs (mean, SD) for the spiked-in hybridization (▲) and polyA+ (▲) controls and for the cRNA degradation (●) and endogenous/housekeeping (○) internal controls. Non-control (background) probesets and the moving mean derived from them are denoted with gray-filled symbols, (•) and (•), respectively. Select spiked-in polyA+ control and RNA degradation probesets are labeled according to the abbreviations in Additional File 1: Supplemental Table S2.

In this evaluation of internal and external controls for assessing microarray performance, it is assumed that these controls act in a manner similar to and consistent with endogenous transcripts in the biological sample when all are assayed with gene expression microarrays. To provide an initial quality assessment of the probeset-specific performance, the variance behavior of the individual probesets of the controls was examined in relation to average signal level across the entire experiment. Similar approaches have been employed to illustrate relationships between probeset signal level and precision metrics in microarray data [2,31-33]. The mean and standard deviation (SD) of the RMA values for all probesets for the 137 hybridizations of the rat experiment is illustrated in Figure 2 for preprocessing with (a) no normalization, (b) quantile normalization and (c) 75th percentile normalization. A comparison of the normalization approaches on this dataset illustrates that the dispersion pattern of the external spike-in controls, as well as the internal Affymetrix controls relative to the mean of the background probesets, are comparable for the (b) quantile normalization and (c) 75th percentile normalization, particularly for intensities greater than 2^8^. The greatest difference observed is for probesets with intensities less than 2^6^, for which the data resembles a "non-normalized" pattern.

The different classes of controls are distinct in terms of the overall variability (SD) across their inherent RMA intensities; this observed difference among the control groups can be used as a screening tool to identify high-quality experimental datasets from the lower-quality or more "noisy" datasets [2]. The experimental dataset shown in Figure 2 is considered of "high-quality", given that the precision for the various controls (as a group) does increase in a systematic fashion with respect to the amount of experimental processing that each group has experienced (Figure 1a). The hybridization controls are expected to have the lowest variability as they are added at the last experimental stage, whereas the polyA+ and endogenous controls are subject to amplification/labeling and degradation steps, respectively, and are thus expected to exhibit greater variability. The overall dispersion of the non-control (background) probesets lends insight into the relative "noise" of the data. For this experiment, the spike-in hybridization controls are at this average or below the average of the non-control probesets whereas the spike-in polyA+ controls are well above this average and near the upper-limit of the background probesets. Notably, the 100 internal endogenous controls or "housekeeping genes" have consistently lower variability across the range of RMA intensities.

The mean/SD plots also reveal the relative precision of individual probesets within a control group relative to other probesets in the experimental dataset. A few of the internal RNA degradation probesets are considerably more variable than both the average background signal and the internal endogenous genes. As shown in Figure 2, the control probesets with the greatest variability include the AFFX_Rat_GAPDH_5_at and AFFX_Rat_GAPDH_M_at RNAd controls (RG5 and RGm, respectively) and the *Dap*, *Thr*, *Phe *and *Lys *polyA+ controls (v/V, w, Y and x/X, respectively). Greater variability, likely attributable to differences in processivity during cRNA labeling, is generally observed for the 5' probesets (denoted with "5"), followed by a moderate level of variability for the probesets that target the middle of the transcript (denoted with "m"). As provided by the quality metrics in the Microarray Centre Quality Assessment (QA) report [26], the majority of hybridizations from this experiment are of acceptable quality, however, several hybridizations exhibit lesser quality and may contribute to the greater variability observed in these probesets. The QA report for Experiment CWTA_0103_01 is included as Additional File 2.

The mean/SD dispersion plots provide an overview of quality through an assessment of probeset-specific performance within the experimental dataset but do not definitively identify particular samples that may be outliers within the experimental dataset. Samples that contribute the greatest amount of variance to the experiment can be resolved through a PCA of the spike-in controls and can be used to identify problems with the discrete sample preparation steps (e.g., hybridization or RNA amplification). Likewise, PCA models of the internal controls can be utilized to verify sample RNA integrity or to account for other sample degradation issues.

### Spike-in Hybridization Controls

**Figure 3:**
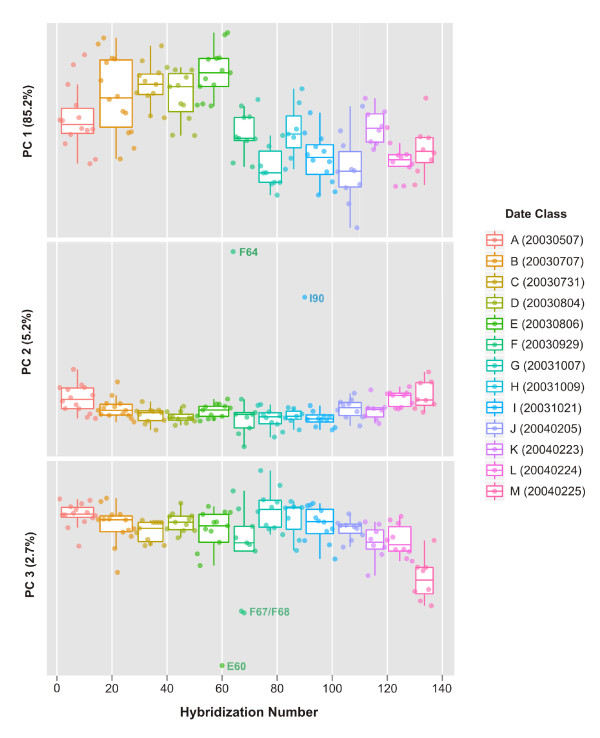
**1-D PCA score plots for the principal components (PC 1, PC 2 and PC 3) for the external spike-in hybridization controls of the rat dataset**. Symbols are color-coded according to the date of hybridization (A - M; see legend) and data from single arrays are overlaid on box plots that summarize the data in each date class. A subset of data points are labeled with both the date class abbreviation (A - M) and the hybridization number (1-137).

In an effort to identify individual arrays that may be problematic, PCA was employed to explore the variability within the spike-in hybridization control dataset. PCA score plots for the first three principal components (PCs) of the hybridization control data subset of the rat CWTA dataset are shown in Figure 3. The data are classified by the date on which a hybridization was performed. For this experiment, a total of 13 hybridization dates were recorded ranging from May 7, 2003 (20030507) to February 25, 2004 (20040225) and are color-coded and denoted by a letters ranging from "A" to "M". The first PC represents roughly 85% of the model variance and highlights a shifting of hybridization intensities between those of date class "E" (20030806) and those of date class "F" (20030929). PC 2 captures an additional 5% of the overall model variance and separates hybridizations (F64 and I90) that have both low quality Scan QC measures (values of 4) and also are outliers with respect to the Normalized Unscaled Standard Error (NUSE) plot [28], shifted log_2 _probe intensities as well as relatively high average array background values and RawQ noise values, the latter of which is a measure of pixel-to-pixel variation among the probesets that is used to calculate the array background [34]. Notably, I90 (NNC2003102101A, Aliquot ID FMTA0048_a; see Table S-1) is a re-hybridization of sample F64 (NNC2003092901A), however there was little improvement to the overall hybridization metrics (i.e., Scan QC, NUSE) Consistent with the relatively high abundance of the biotin-labeled spike-in controls, the scores for PC 2 and PC 3 (< 3% variance) separates hybridizations (F67, F68 and E60) that have relatively low quality Scan QC measures (3 or 4) and have more moderate-to-high average array background values and RawQ values.

The Q residuals of the PCA model (Additional File 1: Supplemental Figure S1) can be used as a diagnostic tool to identify hybridizations that have unusual variation (those that reside outside the PCA model space). In addition, Hotelling T^2 ^values can be used to identify samples that are outliers and that might possess relatively high leverage along the principal axis of the model, analogous to the end points of a linear regression model. The Q residuals in Supplemental Figure S1(a) highlight hybridization B22, which has also been flagged as potential outlier by the NUSE plot. Hotelling T^2 ^values consistently highlight hybridizations F64, E60, I90, F68 for which scanner QC measures have been denoted as problematic (values of 3 or 4).

### Spike-in PolyA+ Controls

A cocktail of RNA controls with artificial polyA+ tails are spiked into each RNA sample over a range of concentrations (Table 2) to monitor the entire sample labeling process. All of the polyA+ controls should be scored as "Present" with signal values: *Lys *>*Phe *>*Dap *>*Thr *>*Trp*. For this experiment, an extremely low correlation (R^2 ^= 0.4498) between the polyA+ spike in concentration and raw signal value observed for hybridization NNC2004020512Aaa (sample J111) as reported in the MiMiR QA report. Correlation values of R^2 ^> 0.95 are expected for typical samples. Outliers such as these are easily identified through an examination of the relative RMA intensities; as an example, the relative RMA intensities for this extreme polyA+ control outlier are shown in Table 2. The difference observed between the average experiment RMA intensity values and that of sample J111 is linearly correlated with log_2 _concentrations for the polyA+ spike-in controls.

**Table 2:** Comparison of polyA+ control RMA values averaged for the entire dataset in contrast with a single outlier sample (J111)

***PolyA+ Control***	***Conc. (ng/μL)***	***log_2 _Conc. (ng/μL)***	***Experiment*** ***RMA values^a, b^***	***Sample J111*** ***RMA values^a^***	***Difference***
			
*Trp*	14.7	3.88	4.69	4.26	0.43
*Thr*	39.1	5.29	8.00	5.06	2.94
*Dap*	108.9	6.77	7.91	4.33	3.58
*Phe*	260.0	8.02	10.23	4.80	5.43
*Lys*	591.1	9.21	11.82	4.61	7.21

^a ^Averaged probeset intensities for each of the polyA+ control group

^b ^Averaged over 137 hybridizations

**Figure 4:**
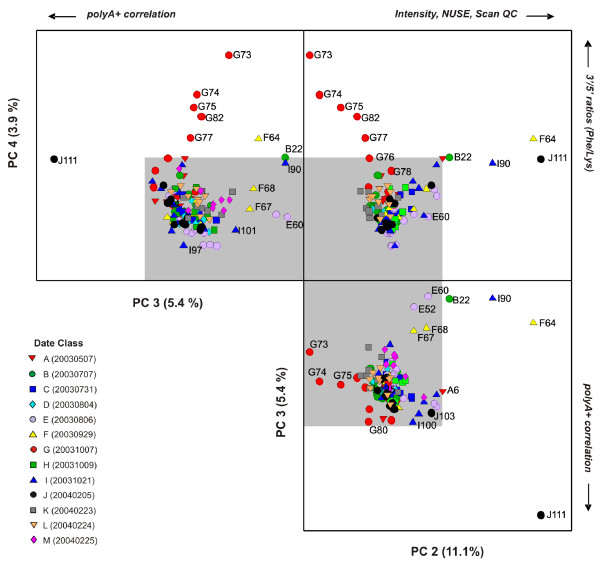
**Unfolded 3-D PCA scores plot (PC 2 × PC 3 × PC 4) for the external spike-in polyA+ controls subset of the rat dataset**. Symbols represent the date class (A - M; see legend).

The PCA model for the polyA+ controls comprises of 4 PCs. The first PC captures the largest variance (76.8%) and primarily separates hybridization J111 from the other 136 hybridizations within the experimental dataset (data in Additional File 1: Supplemental Figure S2(a)). PCs 2, 3 and 4 describe the remaining 20% of variance captured for this model and illustrate more subtle patterns of spike-in polyA+ control quality (Figure 4) that are not readily seen by examining the relative intensities of the controls alone. An unfolded 3-dimensional PCA scores plot of these lower PCs illustrates the various outlying hybridizations that correspond to definitive quality control parameters associated with both assay and hybridization performance. PC 2 (11% of variance) separates hybridizations with the most extreme differences in probe intensities and array background (F64, the I90 re-hybridization of F64, and B22) whereas PC 3 has a primary contribution from the polyA+ control level differences observed for hybridization J111. PC 4 (≈ 4% of variance) uniquely identifies hybridizations conducted on Date "G" (20031007) for which the 3'/5' ratios for the *Phe *and *Lys *polyA+ controls are substantially above the Affymetrix-defined tolerance ratio of 3, which is usually indicative of either insufficient labeling efficiency or poor sample quality. For example, the hybridizations denoted as G73, G74, G75, G82 and G77 had 3'/5' ratios for the relatively high concentration *Phe *polyA+ control of 30.32, 18.91, 11.10, 6.70 and 6.82, respectively.

The J111 outlier can be also identified in the high Hotelling T^2 ^values for the overall model (Additional File 1: Supplemental Figure S2(b)). The loadings for PC 1 have comparable contributions from probesets (X/x, Y/y, V/v, and W/w) that represent the four polyA+ controls (*Lys, Phe, Dap *and *Thr*) (Figure S-2(c)). This result is consistent with the obvious difference in RMA intensity; the log_2 _probe intensities for these four polyA+ controls for hybridization J111 were several orders of magnitude lower when compared to the other hybridizations in the experiment. In contrast, the log_2 _intensities for the *Trp *polyA+ control probesets (Z5, Zm and Z3) were relatively small relative to the overall experiment (median z-score of 0.7). Consistent with the observed intensity data, these probesets have a low contribution to the loadings for the PC 1. In addition, the probeset loading pattern of 5'-middle - 3' trend as observed for the higher concentration controls (*Lys *and *Phe *in Additional File 1: Supplemental Figure S2(c)) indicates that the 5' probeset signals carry more of the variance of the dataset. This is likely attributable to low processivity in the in vitro transcription reaction used to synthesis the polyA+ controls (which proceed in the 3' to 5' direction).

### Internal RNA Degradation and Endogenous Controls

**Figure 5:**
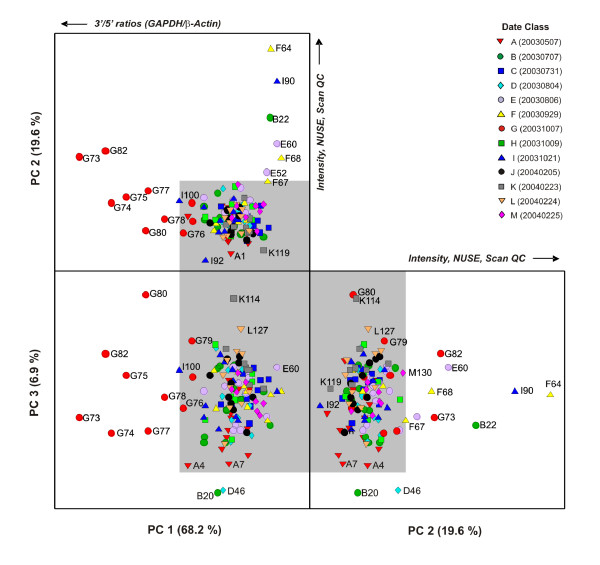
**Unfolded 3-D PCA scores plot (PC 1 × PC 2 × PC 3) for the internal cRNA degradation controls subset of the single Rat dataset**. Symbols as Figure 4.

The PCA model results for the Affymetrix-designated RNA degradation internal control data (Figure 5) illustrate a complementary pattern to the PCA results obtained for the polyA+ external spike-in control dataset but with some subtle differences. For this dataset, the primary contribution of the RNA degradation is realized in the first component of the model (PC 1) followed by the separation of hybridizations that differ in log_2 _probe intensities and overall array quality in the subsequent PCs (2 and 3). This is observed for the group of flagged hybridizations for elevated 3'/5' ratios for GAPDH and/or β-Actin controls (G73, G74, G82, G75, G80, G78 and G77, and to a lesser extent G79, I100 and A4) that are separated in PC 1 and represent 68% of the model variance. Likewise, the major variables that contribute to the loadings for PC 1 correspond to the 5'-end and middle-segments of the Affymetrix GAPDH and β-Actin probesets (RG5, RGm, RbAct5, RbActm; see Additional File 1: Supplemental Figure S3(b)). Hybridizations that correspond to shifted log_2 _probe intensities and elevated NUSE values (F64, I90, B22) are separated on PC 2. Notably, hybridizations B20 and D46 are partially separated from the other hybridizations on PC 3 (≈ 7%), the former of which has a slight indication of cRNA degradation (3'/5' ratio of 3.16 for β-Actin) but it is unclear how D46 (hybridization ID NNC2003070706Aaa) is different from the others with regards to the Affymetrix cRNA degradation internal controls. In all, the PC 1 × PC 2 × PC 3 scores profile as illustrated in Figure 5 represents ≈ 95% of the total model variance.

**Figure 6:**
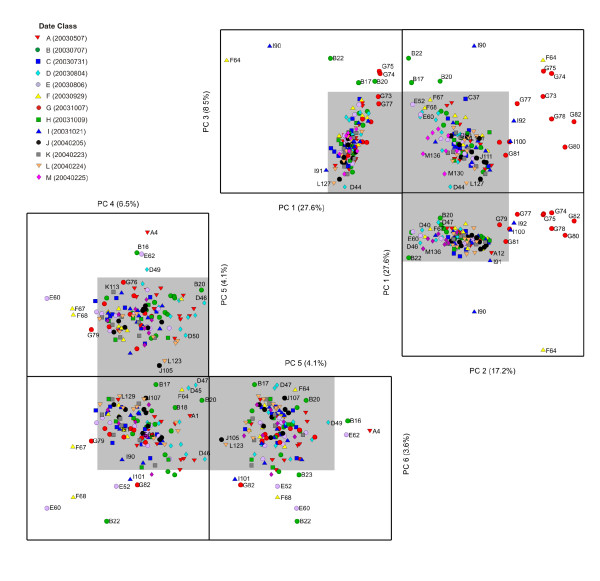
**Two sets of unfolded 3-D PCA scores plot (PC 1 × PC 2 × PC 3 and PC 4 × PC 5 × PC 6) for the endogenous controls from the rat dataset**. Symbols as Figures 4 and 5.

In contrast to the RNA degradation control dataset, the PC 1 × PC 2 × PC 3 scores profile for the PCA model of the endogenous control data (comprised of 100 Affymetrix-identified "housekeeping genes") capture only 53% of the total model variance, with the remainder dispersed among subsequent PCs (Figure 6). The PC 1 × PC 2 × PC 3 profile does, however, have some similarities to the observed patterns for both the external polyA+ and the internal RNA degradation control PCA models. The sample F64 and its I90 rehybridization are present as outliers in PC 1 as is the group of hybridizations (G73, G74, G75, G77, G78, G80, G82, I100) that have been flagged for elevated 3'/5' ratios in PC 2. Notably, PC 3 (8.5% variance) contains additional samples from the Date "B" group (B17, B20), for which the variance contribution is not apparent. The samples that were considered outliers with respect to hybridization and/or scanning issues (F67, F68, E60) are indistinguishable in the PC 1 × PC 2 × PC 3 profile, but are apparent in the lower PC profile (PC 4 × PC 5 × PC 6 layout within Figure 6). Sample J111 is not identified as an outlier within either the internal RNA degradation or endogenous control PCA models; this hybridization is only deemed as an outlier through the polyA+ control model (Figure 4) as its only significant variance is measured via the probesets attributable to the four polyA+ controls (*Lys, Phe, Dap *and *Thr*). This exemplifies the utility of controls that probe data quality at multiple stages in data generation (Figure 1a).

## Conclusions

Different types of controls provide distinct levels of data quality information that can be readily resolved through principal component analysis. A layered PCA modeling of the four classes of controls (spike-in hybridization, spike-in polyA+, internal RNA degradation, endogenous or "housekeeping genes") is valuable for evaluating data quality at a number of stages within the experiment (e.g., hybridization, RNA amplification). The variance at each stage, whether spike-in or internally present, provides complementary information on data quality to those provided by the QA/QC metrics.

This work supports the use of both external and internal control data to assess the technical quality of microarray experiments. In the results presented here, using a layered PCA approach, we find that both the external and internal controls carry with them the critical information about technical performance that is consistent with whole-array quality assessment. This information is obtained for every sample generated using spike-in controls and permits assessment of technical performance for each array. This study is thus a key element in our efforts to develop control methods, materials and designs that support the use of genome-scale data with confidence. Furthermore, these results validate the proposal to use such controls with large data sets generated on multiple platforms or with other multiplexed technology applications.

## Abbreviations

cRNA: copy RNA; CSC/IC: Clinical Sciences Centre/Imperial College; ERCC: External RNA Controls Consortium; MAQC: MicroArray Quality Control; MiMiR: Microarray data Mining Resource; NUSE: Normalized Unscaled Standard Error; PCA: Principal Component Analysis; polyA+: polyadenylated; QA/QC: Quality Assurance/Quality Control; RawQ: Noise attributed to both the scanner and sample quality; RMA: Robust Multichip Average; RMSEC: Root Mean Square Error of Calibration; RMSECV: Root Mean Square Error of Cross Validation; SD: Standard Deviation.

## Competing interests

The authors declare that they have no competing interest.

## Authors' contributions

HCC and LG designed and implemented the spike-in controls for use in the MiMiR experimental protocols in addition to the experimental quality control assessment metrics. HCC, LG and MLS conceived of this study's approach to the analysis of these data and participated in the study design and coordination. DLD developed a prototype for data analysis and display. KAL created the datasets, performed the data preprocessing and chemometric analysis. All authors were involved in the data interpretation. KAL and MLS drafted the manuscript and all authors read and approved the final manuscript.

## Supplementary Material

Additional file 1 **contains the PCA model results include both diagnostic Q/Hotelling T^2 ^plots and loadings plots for the spike-in hybridization and polyA+ control data and the internal cRNA degradation control data subsets in Supplemental Figures S1, S2, and S3 respectively**. Additionally, two supplemental tables are provided to aid in the data interpretation within the manuscript. These include Table S1 that provides condensed annotation information for the single Rat experiment and Table S2 that lists the probe set identifiers for spike-in hybridization and polyA+ controls together with the internal Affymetrix cRNA degradation (RNAd) and endogenous controls for the RAE230A array. Click here for file

Additional file 2 **contains a copy of the Microarray Centre Quality Assessment of Affymetrix Data for EXP_CWTA_0103_01 comprising 138 hybridizations on Rat Expression Set 230A Arrays. Hybridization HFB2003080611Aaa listed in the QA report was excluded from the PCA dataset as the full annotation information was not available at the time of this study**. Click here for file
