# Supplementary material for: Exploring the use of internal and externalcontrols for assessing microarray technical performance
Source: BMC Res Notes. 2010 Dec 28;3:349. doi: 10.1186/1756-0500-3-349 (PMC3020182; doi:10.1186/1756-0500-3-349)
Supplement: Additional file 1 — contains the PCA model results include both diagnostic Q/Hotelling T2 plots and loadings plots for the spike-in hybridization and polyA+ control data and the internal cRNA degradation control data subsets in Supplemental Figures S1, S2, and S3 respectively. Additionally, two supplemental tables are provided to aid in the data interpretation within the manuscript. These include Table S1 that provides condensed annotation information for the single Rat experiment and Table S2 that lists the probe set identifiers for spike-in hybridization and polyA+ controls together with the internal Affymetrix cRNA degradation (RNAd) and endogenous controls for the RAE230A array. [file 1756-0500-3-349-S1.PDF]

ADDITIONAL FILE 1

for

Exploring the use of internal and external controls for assessing microarray technical performance

Katrice A. Lippa,[1,*] David L. Duewer,[1] Marc L. Salit,[1] Laurence Game[2] and Helen C. Causton[2,3]

[1]Chemical Science and Technology Laboratory
National Institute of Standards and Technology (NIST)
Gaithersburg, Maryland 20899 USA

[2]MRC Clinical Sciences Centre (CSC)
Imperial College Microarray Centre
Hammersmith Hospital Campus
London W12 0NN, United Kingdom

[3]Department of Biological Sciences
Columbia University
New York, NY 10027 USA

Additional File 1 contains supplemental figures for the PCA model results include both diagnostic Q/Hotelling T^2 plots and loadings plots for the spike-in hybridization and polyA+ control data and the internal cRNA degradation control data subsets in Supplemental Figures S1, S2, and S3 respectively. Additionally, two supplemental tables are provided to aid in the data interpretation within the manuscript. These include Table S1 that provides condensed annotation information for the single Rat experiment and Table S2 that lists the probe set identifiers for spike-in hybridization and polyA+ controls together with the internal Affymetrix cRNA degradation (RNAd) and endogenous controls for the RAE230A array.

(a)

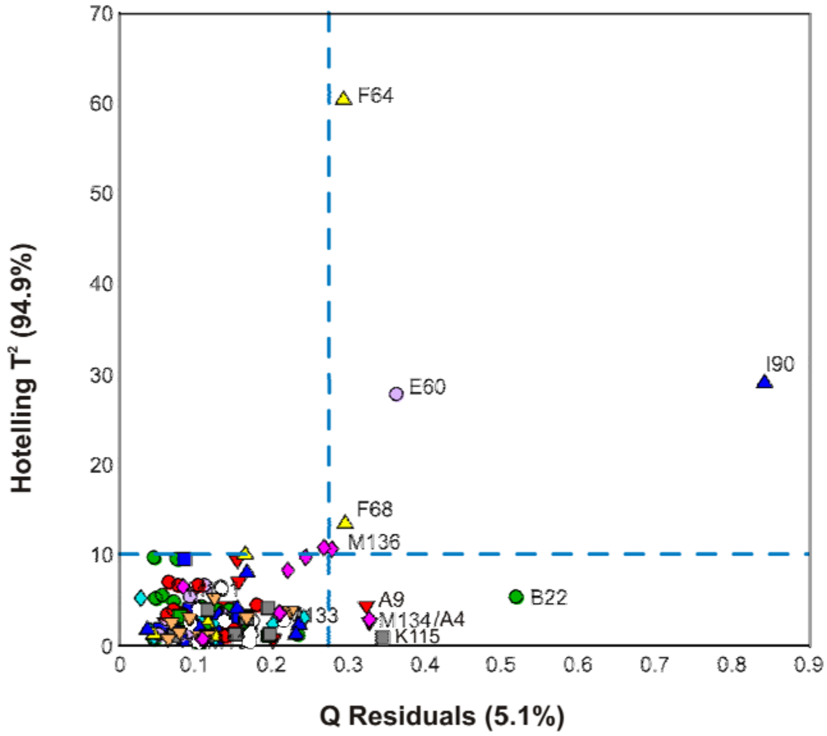

(b)

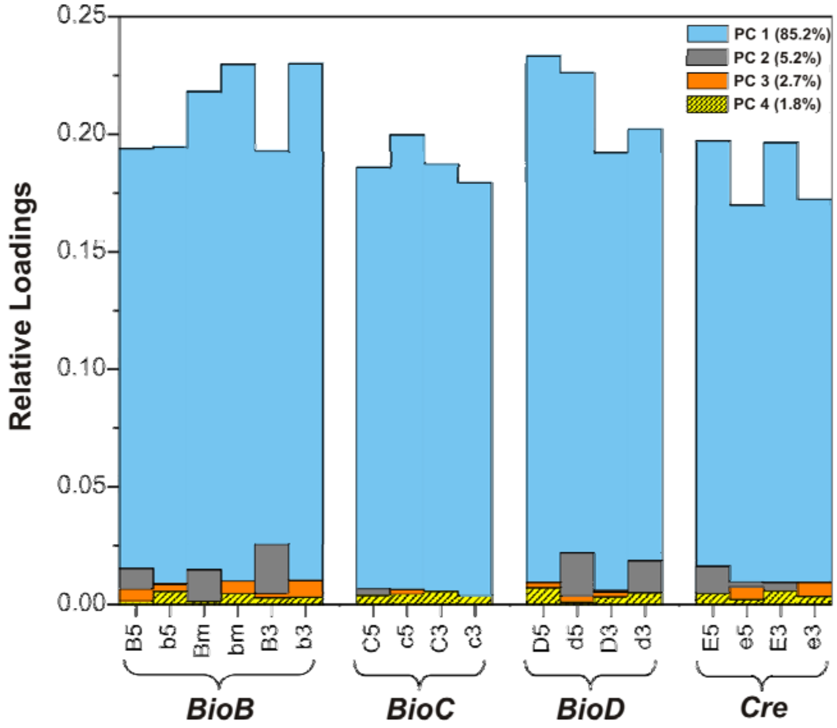

Supplemental Figure S1. Spiked-in hybridization control PCA model. (a) Diagnostic Q/Hotelling T^2 plot, the dashed blue lines represent the 95% confidence limits. (b) Loadings plot, the absolute loading of each variable (probe set) is normalized to the % variance captured per PC (as displayed in legend).

(a)

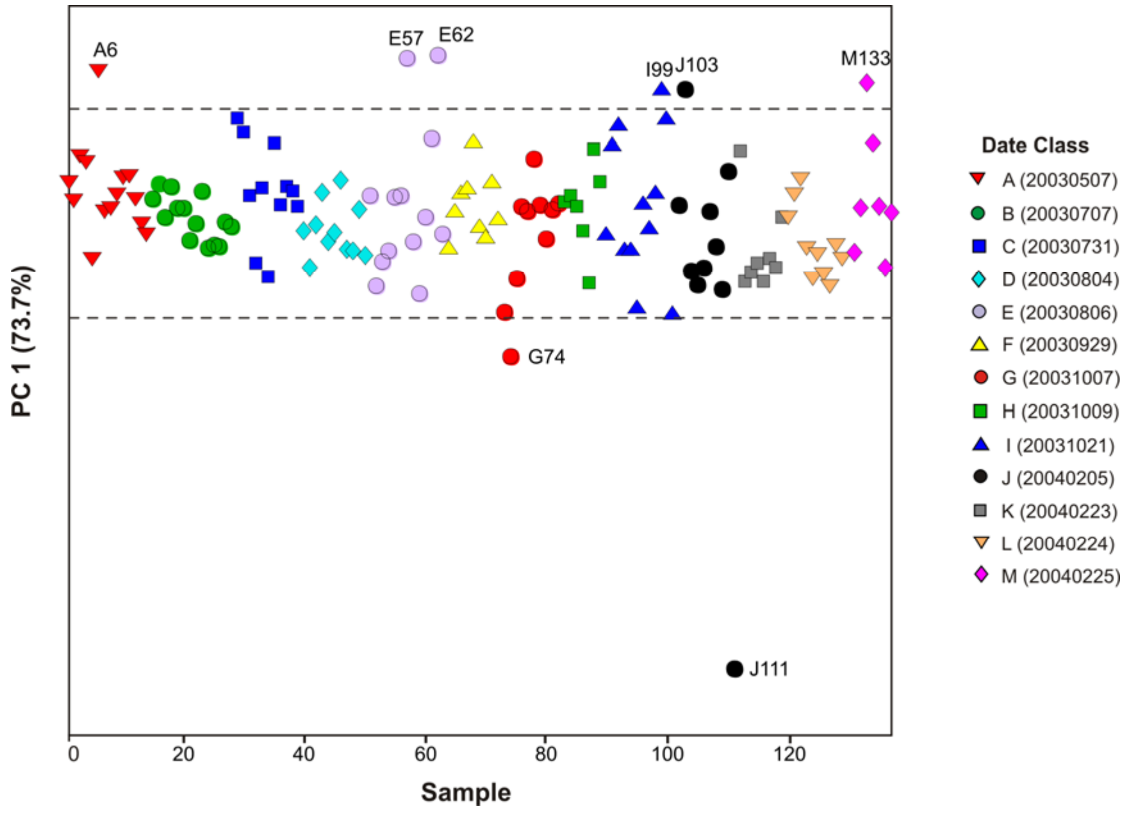

Supplemental Figure S2. Spiked-in polyA+ control PCA model supplemental results. (a) 1-D PCA scores plot (PC 1). Symbols represent the date class (A – M; see legend).

(b)

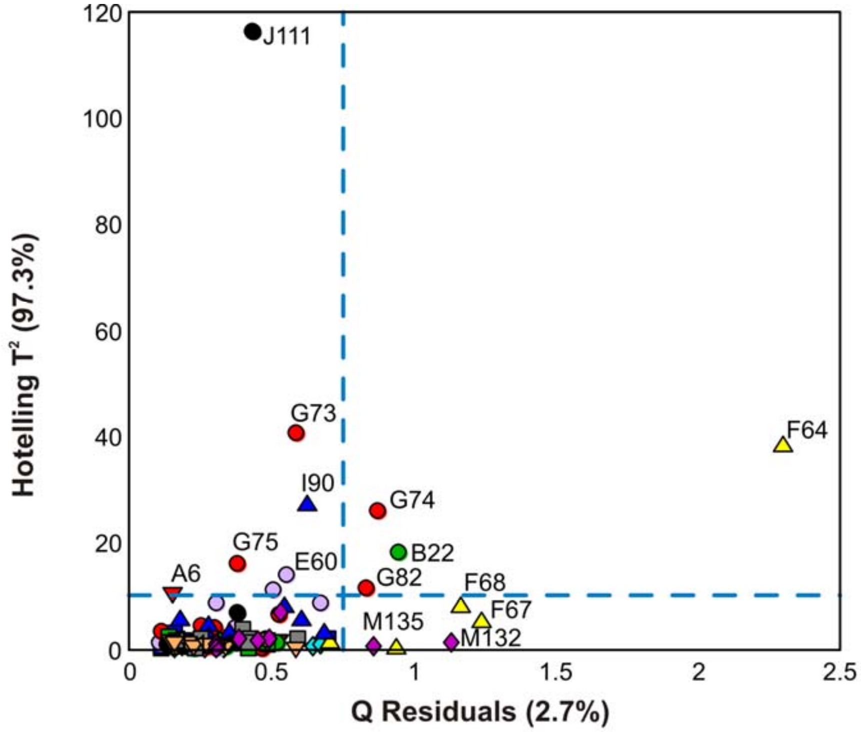

(c)

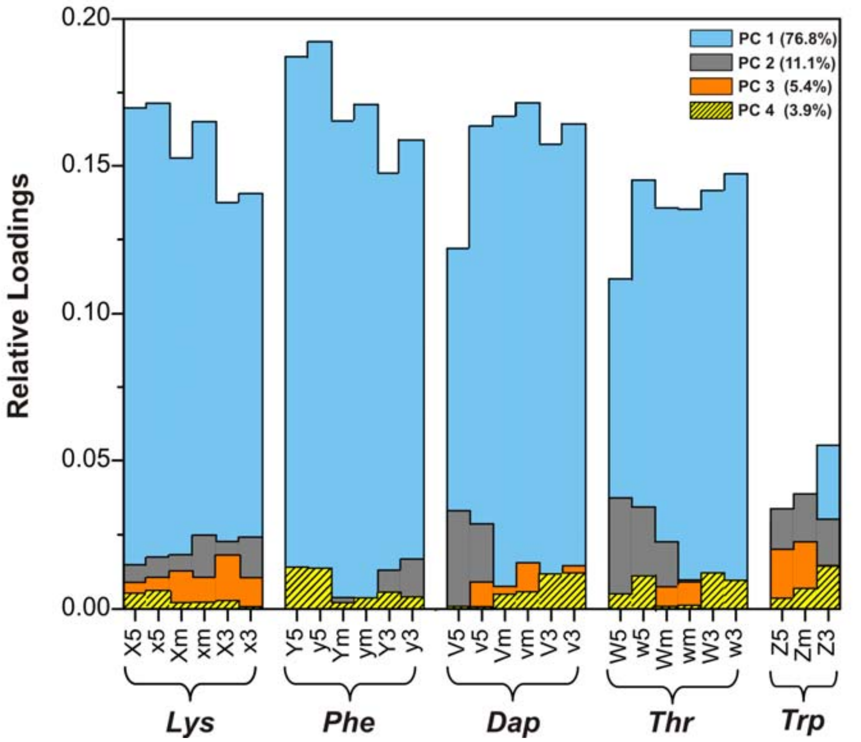

Supplemental Figure S2 (cont'd). Spiked-in polyA+ control PCA model supplemental results. (b) Diagnostic Q/Hotelling T^2 plot with the dashed blue lines represent the 95% confidence limits. (c) Loadings plot for which the absolute loading of each variable (probe set) is normalized to the % variance captured per PC (as displayed in legend).

(a)

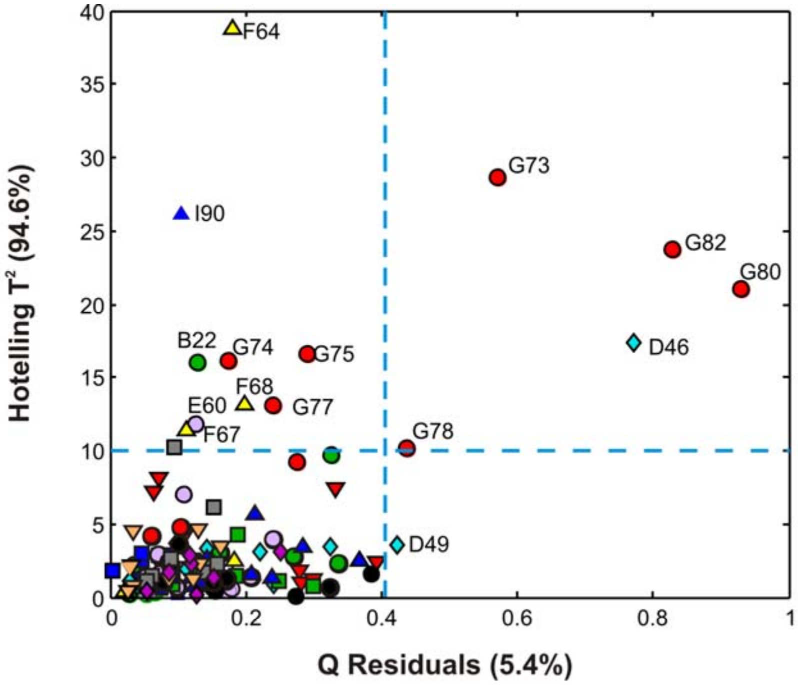

(b)

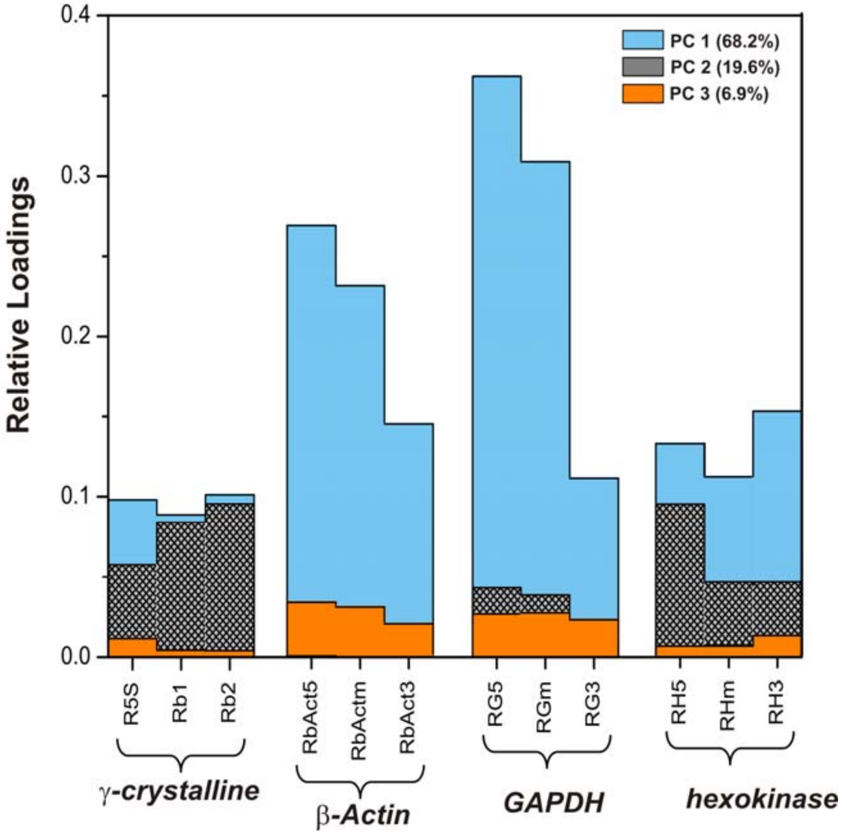

Supplemental Figure S3. Internal cRNA degradation control PCA model supplemental results. (a) Diagnostic Q/Hotelling T^2 plot with the dashed blue lines represent the 95% confidence limits. (b) Loadings plot for which the absolute loading of each variable (probe set) is normalized to the % variance captured per PC (as displayed in legend).

Supplemental Table S1. Condensed annotation information for the single Rat experiment comprised of 137 hybridizations on a RAE230A array. Full annotation information for this experiment (EXP_CWTA_0103_01; Array Express ID E-MIMR-222) is available at: http://microarray.csc.mrc.ac.uk. The table is sorted by Hybridization ID to complement the QA report provided in Appendix A.

Hybridization ID[a]	PCA Sample ID	Scan QC	cRNA QC	tRNA QC	MiMiR/ (bio)sample ID[b]	Aliquot ID	Labeling protocol[c]	Additional Information
FFR2004022301A	K112	1	1	1	BN-Lx-6_f	FMTA0079_a	Std - Affy	
FFR2004022302A	K113	1	1	1	SHR/Ola-8_f	FMTA0080_a	Std - Affy	
FFR2004022303A	K114	1	1	1	RI 01-5_f	FMTA0081_a	Std - Affy	
FFR2004022304A	K115	1	1	1	RI 02-3_f2	FMTA0082_a	Std - Affy	
FFR2004022306A	K116	1	2	2	RI 05-4_f	FMTA0084_a	Std - Affy	
FFR2004022308A	K117	1	1	1	RI 05c-5_f	FMTA0086_a	Std - Affy	
FFR2004022309A	K118	1	1	3	RI 06c-4_f	FMTA0087_a	Std - Affy	
FFR2004022310A	K119	1	1	1	RI 09c-5_f	FMTA0088_a	Std - Affy	
FFR2004022501A	M130	2	2	1	RI 12c-5_f	CWTA0094_a	Std - Affy	
FFR2004022502A	M131	2	1	na	RI 17-2_f	CWTA0095_a	Std - Affy	
FFR2004022503A	M132	1	1	na	RI 03c-4_f	FMTA0099_a	Std - Affy	
FFR2004022504A	M133	1	3	na	RI 13c-3_f	FMTA0100_a	Std - Affy	
FFR2004022505A	M134	1	2	na	RI 15-6_f	FMTA0101_a	Std - Affy	
FFR2004022506A	M135	1	1	1	RI 24-5_f	FMTA0102_a	Std - Affy	
FFR2004022507A	M136	1	1	na	RI 25-3_f	FMTA0103_a	Std - Affy	
FFR2004022508A	M137	1	1	1	RI 25-4_f	FMTA0104_a	Std - Affy	
HFB2003050701A	A1	1	3	1	SHR/Ola-1_f	CWTA0026_a	Std - Affy	
HFB2003050702A	A2	1	1	1	SHR/Ola-2_f	CWTA0027_a	Std - Affy	
HFB2003050703A	A3	1	1	1	BN-Lx-1_f	CWTA0028_a	Std - Affy	
HFB2003050705A	A4	1	3	1	RI 01-1_f	CWTA0030_a	Std - Affy	
HFB2003050706A	A5	1	1	2	RI 02-2_f	CWTA0031_a	Std - Affy	
HFB2003050707A	A6	1	3	1	RI 2c-1_f	CWTA0032_a	Std - Affy	
HFB2003050708A	A7	1	2	1	RI 03-1_f	CWTA0033_a	Std - Affy	
HFB2003050709A	A8	1	1	1	RI 05c-2_f	CWTA0034_a	Std - Affy	
HFB2003050710A	A9	1	2	1	RI 06c-1_f	CWTA0035_a	Std - Affy	
HFB2003050711A	A10	1	3	1	RI 07-1_f	CWTA0036_a	Std - Affy	
HFB2003050712A	A11	1	2	1	RI 07-2_f	CWTA0037_a	Std - Affy	
HFB2003050713A	A12	1	1	1	RI 09c-2_f	CWTA0038_a	Std - Affy	
HFB2003050714A	A13	1	1	1	RI 10-2_f	CWTA0039_a	Std - Affy	
HFB2003050715A	A14	1	1	1	RI 10c-2_f	CWTA0040_a	Std - Affy	

HFB2003073101A	C29	3	1	na	RI 05-2_f	CWTA0041_a	Std - Affy	
HFB2003073102A	C30	2	1	na	RI 04-2_f	CWTA0042_a	Std - Affy	
HFB2003073103A	C31	2	1	na	RI 04-1_f	CWTA0043_a	Std - Affy	
HFB2003073104A	C32	3	1	na	RI 11c-2_f	CWTA0044_a	Std - Affy	
HFB2003073105A	C33	3	2	na	RI 02-1_f	CWTA0045_a	Std - Affy	
HFB2003073106A	C34	3	2	na	RI 13c-2_f	CWTA0046_a	Std - Affy	
HFB2003073107A	C35	3	2	na	RI 09c-1_f	CWTA0047_a	Std - Affy	
HFB2003073108A	C36	3	1	na	RI 03-2_f	CWTA0048_a	Std - Affy	
HFB2003073109A	C37	2	1	na	RI 03c-1_f	CWTA0049_a	Std - Affy	
HFB2003073110A	C38	2	1	na	RI 03c-2_f	CWTA0050_a	Std - Affy	
HFB2003073111A	C39	3	1	na	RI 11c-1_f	CWTA0051_a	Std - Affy	
HFB2003080401A	D40	3	2	na	RI 18-1_f	CWTA0052_a	Std - Affy	
HFB2003080402A	D41	3	1	na	RI 23-1_f	CWTA0053_a	Std - Affy	
HFB2003080403A	D42	3	1	na	RI 20-2_f	CWTA0054_a	Std - Affy	
HFB2003080404A	D43	2	1	na	RI 17-1_f	CWTA0055_a	Std - Affy	
HFB2003080406A	D44	3	2	na	RI 21-2_f	CWTA0057_a	Std - Affy	
HFB2003080407A	D45	3	2	na	RI 15-1_f	CWTA0058_a	Std - Affy	
HFB2003080408A	D46	3	2	na	RI 08c-2_f	CWTA0059_a	Std - Affy	
HFB2003080409A	D47	3	1	na	RI 15-2_f	CWTA0060_a	Std - Affy	
HFB2003080410A	D48	3	1	na	RI 12c-2_f	CWTA0061_a	Std - Affy	re-hybridized (I94)
HFB2003080411A	D49	3	1	na	RI 20-1_f	CWTA0062_a	Std - Affy	
HFB2003080412A	D50	3	1	na	RI 18-2_f	CWTA0063_a	Std - Affy	
HFB2003080601A	E51	3	1	na	RI 10c-1_f	CWTA0064_a	Std - Affy	
HFB2003080602A	E52	3	na	na	RI 24-2_f	CWTA0065_a	Std - Affy	re-hybridized (I95)
HFB2003080603A	E53	3	1	na	RI 23-2_f	CWTA0066_a	Std - Affy	
HFB2003080604A	E54	3	3	na	RI 31-2_f	CWTA0067_a	Std - Affy	
HFB2003080605A	E55	1	2	na	RI 22-2_f	CWTA0068_a	Std - Affy	
HFB2003080606A	E56	2	1	na	RI 27-1_f	CWTA0069_a	Std - Affy	re-hybridized (I96)
HFB2003080607A	E57	2	1	na	RI 13c-1_f	CWTA0070_a	Std - Affy	
HFB2003080608A	E58	2	1	na	RI 26-2_f	CWTA0071_a	Std - Affy	
HFB2003080609A	E59	2	1	na	RI 26-1_f	CWTA0072_a	Std - Affy	
HFB2003080610A	E60	3	1	na	RI 31-1_f	CWTA0073_a	Std - Affy	re-hybridized (I97)
HFB2003080612A	E61	2	2	na	RI 29-2_f	CWTA0075_a	Std - Affy	
HFB2003080613A	E62	1	1	na	RI 01-2_f	CWTA0076_a	Std - Affy	
HFB2003080614A	E63	2	3	na	RI 29-1_f	CWTA0077_a	Std - Affy	
NNC2003070701A	B15	2	1	1	BN-Lx-3_f	FMTA0023_a	Std - Affy	
NNC2003070702A	B16	2	1	1	RI 01-4_f	FMTA0024_a	Std - Affy	
NNC2003070703A	B17	1	1	1	RI 02-4_f	FMTA0025_a	Std - Affy	

NNC2003070704A	B18	2	1	1	RI 2c-3_f	FMTA0026_a	Std - Affy	
NNC2003070705A	B19	1	1	1	RI 2c-4_f	FMTA0027_a	Std - Affy	
NNC2003070706A	B20	2	1	1	RI 03-4_f	FMTA0028_a	Std - Affy	
NNC2003070707A	B21	2	1	1	RI 08c-3_f	FMTA0029_a	Std - Affy	
NNC2003070708A	B22	3	1	na	RI 08c-4_f	FMTA0030_a	Std - Affy	
NNC2003070709A	B23	3	2	1	RI 10-3_f	FMTA0031_a	Std - Affy	
NNC2003070710A	B24	3	2	1	RI 10c-3_f	FMTA0032_a	Std - Affy	
NNC2003070711A	B25	3	1	1	RI 11c-3_f	FMTA0033_a	Std - Affy	
NNC2003070712A	B26	3	na	na	RI 11c-4_f	FMTA0034_a	Std - Affy	
NNC2003070713A	B27	3	2	1	RI 12c-3_f	FMTA0035_a	Std - Affy	
NNC2003070714A	B28	3	2	1	RI 12c-4_f	FMTA0036_a	Std - Affy	
NNC2003092901A	F64	4	na	na	RI 02-3_f	FMTA0048_a	Std - Affy	re-hybridized (I90)
NNC2003092903A	F65	3	1	1	RI 04-3_f	FMTA0050_a	Std - Affy	
NNC2003092904A	F66	1	2	1	RI 04-4_f	FMTA0051_a	Std - Affy	
NNC2003092905A	F67	4	2	1	RI 05-3_f	FMTA0052_a	Std - Affy	
NNC2003092906A	F68	4	2	1	RI 05c-3_f	FMTA0053_a	Std - Affy	
NNC2003092907A	F69	2	3	1	RI 07-3_f	FMTA0054_a	Std - Affy	
NNC2003092908A	F70	2	2	1	RI 07-4_f	FMTA0055_a	Std - Affy	
NNC2003092909A	F71	1	3	na	RI 13c-4_f	FMTA0056_a	Std - Affy	
NNC2003092910A	F72	1	2	na	RI 03c-3_f	FMTA0057_a	Std - Affy	
NNC2003100701A	G73	3	3	na	RI 17-3_f	FMTA0058_a	Std - Affy	
NNC2003100702A	G74	3	3	na	RI 18-3_f	FMTA0059_a	Std - Affy	
NNC2003100703A	G75	3	3	na	RI 18-4_f	FMTA0060_a	Std - Affy	
NNC2003100705A	G76	2	3	na	RI 21-3_f	FMTA0062_a	Std - Affy	
NNC2003100706A	G77	3	3	na	RI 22-3_f	FMTA0063_a	Std - Affy	
NNC2003100707A	G78	3	3	na	RI 22-4_f	FMTA0064_a	Std - Affy	
NNC2003100708A	G79	3	3	na	RI 23-3_f	FMTA0065_a	Std - Affy	
NNC2003100710A	G80	2	3	na	RI 26-3_f	FMTA0067_a	Std - Affy	
NNC2003100711A	G81	1	3	na	RI 27-4_f	FMTA0068_a	Std - Affy	
NNC2003100712A	G82	3	3	na	RI 20-3_f	FMTA0069_a	Std - Affy	
NNC2003100901A	H83	2	2	na	SHR/Ola-7_f	FMTA0070_a	Std - Affy	
NNC2003100902A	H84	2	1	na	RI 08c-4_f	FMTA0071_a	Std - Affy	
NNC2003100904A	H85	1	2	na	RI 24-3_f	FMTA0073_a	Std - Affy	
NNC2003100905A	H86	1	1	na	RI 31-3_f	FMTA0074_a	Std - Affy	
NNC2003100906A	H87	2	1	na	RI 31-4_f	FMTA0075_a	Std - Affy	
NNC2003100907A	H88	3	3	na	RI 06c-6_f	FMTA0076_a	Std - Affy	
NNC2003100909A	H89	1	1	1	BN-Lx-5_f	FMTA0047_c	Std - Affy	
NNC2003102101A	I90	4	na	na	RI 02-3_f	FMTA0048_a	Std - Affy	rehybridization of F64

NNC2003102102A	I91	2	1	1	RI 23-4_f	FMTA0072_a	Std - Affy	
NNC2003102103A	I92	1	2	1	RI 03-3_f	FMTA0077_a	Std - Affy	
NNC2003102104A	I93	1	3	1	RI 29-4_f	FMTA0078_a	Std - Affy	
NNC2003102105A	I94	2	1	na	RI 12c-2_f	CWTA0061_c	Std - Affy	rehybridization of D48
NNC2003102106A	I95	2	na	na	RI 24-2_f	CWTA0065_c	Std - Affy	rehybridization of E52
NNC2003102107A	I96	1	1	na	RI 27-1_f	CWTA0069_c	Std - Affy	rehybridization of E56
NNC2003102108A	I97	1	1	na	RI 31-1_f	CWTA0073_c	Std - Affy	rehybridization of E60
NNC2003102109A	I98	2	1	1	RI 25-1_f	CWTA0078_a	Std - Affy	
NNC2003102110A	I99	2	3	1	RI 27-2_f	CWTA0079_a	Std - Affy	
NNC2003102111A	I100	1	3	1	RI 05c-1_f	CWTA0080_a	Std - Affy	
NNC2003102112A	I101	3	1	1	RI 05-1_f	CWTA0081_a	Std - Affy	
NNC2004020501A	J102	2	2	3	RI 06c-5_f	CWTA0082_a	Std - Affy	
NNC2004020502A	J103	2	2	1	RI 2c-5_f	CWTA0083_a	Std - Affy	
NNC2004020503A	J104	2	1	2	BN-Lx-2_f	CWTA0084_a	Std - Affy	
NNC2004020504A	J105	2	1	1	RI 08c-5_f	CWTA0085_a	Std - Affy	
NNC2004020507A	J106	2	1	1	RI 10-5_f	CWTA0088_a	Std - Affy	
NNC2004020508A	J107	2	1	1	RI 25-5_f	CWTA0089_a	Std - Affy	
NNC2004020509A	J108	2	2	na	RI 24-1_f	CWTA0090_a	Std - Affy	
NNC2004020510A	J109	2	1	1	SHR/Ola-6_f	CWTA0091_a	Std - Affy	
NNC2004020511A	J110	2	1	1	RI 22-1_f	CWTA0092_a	Std - Affy	
NNC2004020512A	J111	2	1	1	RI 21-1_f	CWTA0093_a	Std - Affy	
NNC2004022401A	L120	2	1	1	RI 09c-4_f	FMTA0089_a	Std - Affy	
NNC2004022402A	L121	1	1	1	RI 10-4_f	FMTA0090_a	Std - Affy	
NNC2004022403A	L122	1	1	1	RI 10c-5_f	FMTA0091_a	Std - Affy	
NNC2004022404A	L123	2	3	1	RI 15-5_f	FMTA0092_a	Std - Affy	
NNC2004022405A	L124	1	1	1	RI 17-4_f	FMTA0093_a	Std - Affy	
NNC2004022406A	L125	2	2	na	RI 20-4_f	FMTA0094_a	Std - Affy	
NNC2004022407A	L126	2	1	1	RI 21-4_f	FMTA0095_a	Std - Affy	
NNC2004022408A	L127	2	1	na	RI 26-4_f	FMTA0096_a	Std - Affy	
NNC2004022409A	L128	1	1	na	RI 27-3_f	FMTA0097_a	Std - Affy	
NNC2004022410A	L129	1	1	1	RI 29-5_f	FMTA0098_a	Std - Affy	

[a] Hybridization ID is coded (as an example, HFB2003050703A) with the analyst's initials (HFB), date of hybridization (20030507), number in the hybridization sequence (03) and array type (A)

[b] Recombinant inbred (RI) rat strains derived from the spontaneously hypertensive rat (SHR/Ola) and Brown Norway congenic carrying polydactylyl-luxate syndrome (BN-Lx)

[c] Labeling protocol: Std - Affy = Standard protocol - ENZO - Affy cleanup

Supplemental Table S2. List of probe set identifiers for spiked-in hybridization and polyA+ controls together with the internal Affymetrix RNA degradation (RNAd) and endogenous controls (XendoC) on the RAE230A array

Control Type	Abbreviation	Description
Spiked-in Hybridization Controls:		
BioB	B5	AFFX-BioB-5_at
BioB	b5	AFFX-r2-Ec-bioB-5_at
BioB	Bm	AFFX-BioB-M_at
BioB	bm	AFFX-r2-Ec-bioB-M_at
BioB	B3	AFFX-BioB-3_at
BioB	b3	AFFX-r2-Ec-bioB-3_at
BioC	C5	AFFX-BioC-5_at
BioC	c5	AFFX-r2-Ec-bioC-5_at
BioC	C3	AFFX-BioC-3_at
BioC	c3	AFFX-r2-Ec-bioC-3_at
BioD	D5	AFFX-BioDn-5_at
BioD	d5	AFFX-r2-Ec-bioD-5_at
BioD	D3	AFFX-BioDn-3_at
BioD	d3	AFFX-r2-Ec-bioD-3_at
Cre	E5	AFFX-CreX-5_at
Cre	e5	AFFX-r2-P1-cre-5_at
Cre	E3	AFFX-CreX-3_at
Cre	e3	AFFX-r2-P1-cre-3_at
Spiked-in PolyA+ Controls:		
Lys	X5	AFFX-LysX-5_at
Lys	x5	AFFX-r2-Bs-lys-5_at
Lys	Xm	AFFX-LysX-M_at
Lys	xm	AFFX-r2-Bs-lys-M_at
Lys	X3	AFFX-LysX-3_at
Lys	x3	AFFX-r2-Bs-lys-3_at
Phe	Y5	AFFX-PheX-5_at
Phe	y5	AFFX-r2-Bs-phe-5_at
Phe	Ym	AFFX-PheX-M_at
Phe	ym	AFFX-r2-Bs-phe-M_at
Phe	Y3	AFFX-PheX-3_at
Phe	y3	AFFX-r2-Bs-phe-3_at
Dap	V5	AFFX-DapX-5_at
Dap	v5	AFFX-r2-Bs-dap-5_at
Dap	Vm	AFFX-DapX-M_at
Dap	vm	AFFX-r2-Bs-dap-M_at
Dap	V3	AFFX-DapX-3_at
Dap	v3	AFFX-r2-Bs-dap-3_at
Thr	W5	AFFX-ThrX-5_at
Thr	w5	AFFX-r2-Bs-thr-5_s_at
Thr	Wm	AFFX-ThrX-M_at
Thr	wm	AFFX-r2-Bs-thr-M_s_at
Thr	W3	AFFX-ThrX-3_at

Thr	w3	AFFX-r2-Bs-thr-3_s_at
Trp	Z5	AFFX-TrpnX-5_at
Trp	Zm	AFFX-TrpnX-M_at
Trp	Z3	AFFX-TrpnX-3_at

RNA degradation (Affymetrix) Controls:

RNAd	R5S	AFFX_rat_5S_rRNA_at
RNAd	RbAct3	AFFX_Rat_beta-actin_3_at
RNAd	RbAct5	AFFX_Rat_beta-actin_5_at
RNAd	RbActm	AFFX_Rat_beta-actin_M_at
RNAd	RG3	AFFX_Rat_GAPDH_3_at
RNAd	RG5	AFFX_Rat_GAPDH_5_at
RNAd	RGm	AFFX_Rat_GAPDH_M_at
RNAd	RH3	AFFX_Rat_Hexokinase_3_at
RNAd	RH5	AFFX_Rat_Hexokinase_5_at
RNAd	RHm	AFFX_Rat_Hexokinase_M_at
RNAd	Rb1	AFFX_ratb1/X12957_at
RNAd	Rb2	AFFX_ratb2/X14115_at

Endogeneous or Normalization Controls:

XendoC	XC1	1367452_at
XendoC	XC2	1367453_at
XendoC	XC3	1367454_at
XendoC	XC4	1367455_at
XendoC	XC5	1367456_at
XendoC	XC6	1367457_at
XendoC	XC7	1367458_at
XendoC	XC8	1367459_at
XendoC	XC9	1367460_at
XendoC	XC10	1367461_at
XendoC	XC11	1367462_at
XendoC	XC12	1367463_at
XendoC	XC13	1367464_at
XendoC	XC14	1367465_at
XendoC	XC15	1367466_at
XendoC	XC16	1367467_at
XendoC	XC17	1367468_at
XendoC	XC18	1367469_at
XendoC	XC19	1367470_at
XendoC	XC20	1367471_at
XendoC	XC21	1367472_at
XendoC	XC22	1367473_at
XendoC	XC23	1367474_at
XendoC	XC24	1367475_at
XendoC	XC25	1367476_at
XendoC	XC26	1367477_at
XendoC	XC27	1367478_at
XendoC	XC28	1367479_at
XendoC	XC29	1367480_at
XendoC	XC30	1367481_at

XendoC	XC31	1367482_at
XendoC	XC32	1367483_at
XendoC	XC33	1367484_at
XendoC	XC34	1367485_at
XendoC	XC35	1367486_at
XendoC	XC36	1367487_at
XendoC	XC37	1367488_at
XendoC	XC38	1367489_at
XendoC	XC39	1367490_at
XendoC	XC40	1367491_at
XendoC	XC41	1367492_at
XendoC	XC42	1367493_at
XendoC	XC43	1367494_at
XendoC	XC44	1367495_at
XendoC	XC45	1367496_at
XendoC	XC46	1367497_at
XendoC	XC47	1367498_at
XendoC	XC48	1367499_at
XendoC	XC49	1367500_at
XendoC	XC50	1367501_at
XendoC	XC51	1367502_at
XendoC	XC52	1367503_at
XendoC	XC53	1367504_at
XendoC	XC54	1367505_at
XendoC	XC55	1367506_at
XendoC	XC56	1367507_at
XendoC	XC57	1367508_at
XendoC	XC58	1367509_at
XendoC	XC59	1367510_at
XendoC	XC60	1367511_at
XendoC	XC61	1367512_at
XendoC	XC62	1367513_at
XendoC	XC63	1367514_at
XendoC	XC64	1367515_at
XendoC	XC65	1367516_at
XendoC	XC66	1367517_at
XendoC	XC67	1367518_at
XendoC	XC68	1367519_at
XendoC	XC69	1367520_at
XendoC	XC70	1367521_at
XendoC	XC71	1367522_at
XendoC	XC72	1367523_at
XendoC	XC73	1367524_at
XendoC	XC74	1367525_at
XendoC	XC75	1367526_at
XendoC	XC76	1367527_at
XendoC	XC77	1367528_at
XendoC	XC78	1367529_at
XendoC	XC79	1367530_at
XendoC	XC80	1367531_at
XendoC	XC81	1367532_at

XendoC	XC82	1367533_at
XendoC	XC83	1367534_at
XendoC	XC84	1367535_at
XendoC	XC85	1367536_at
XendoC	XC86	1367537_at
XendoC	XC87	1367538_at
XendoC	XC88	1367539_at
XendoC	XC89	1367540_at
XendoC	XC90	1367541_at
XendoC	XC91	1367542_at
XendoC	XC92	1367543_at
XendoC	XC93	1367544_at
XendoC	XC94	1367545_at
XendoC	XC95	1367546_at
XendoC	XC96	1367547_at
XendoC	XC97	1367548_at
XendoC	XC98	1367549_a_at
XendoC	XC99	1367550_a_at
XendoC	XC100	1367551_a_at
