# Supplementary material for: Exploring the use of internal and externalcontrols for assessing microarray technical performance
Source: BMC Res Notes. 2010 Dec 28;3:349. doi: 10.1186/1756-0500-3-349 (PMC3020182; doi:10.1186/1756-0500-3-349)
Supplement: Additional file 2 — contains a copy of the Microarray Centre Quality Assessment of Affymetrix Data for EXP_CWTA_0103_01 comprising 138 hybridizations on Rat Expression Set 230A Arrays. Hybridization HFB2003080611Aaa listed in the QA report was excluded from the PCA dataset as the full annotation information was not available at the time of this study. [file 1756-0500-3-349-S2.PDF]

ADDITIONAL FILE 2

for

Exploring the use of internal and external controls for assessing microarray technical performance

Katrice A. Lippa,[1,*] David L. Duewer,[1] Marc L. Salit,[1] Laurence Game[2] and Helen C. Causton[2,3]

[1]Chemical Science and Technology Laboratory
National Institute of Standards and Technology (NIST)
Gaithersburg, Maryland 20899 USA

[2]MRC Clinical Sciences Centre (CSC)
Imperial College Microarray Centre
Hammersmith Hospital Campus
London W12 0NN, United Kingdom

[3]Department of Biological Sciences
Columbia University
New York, NY 10027 USA

Additional File 2 contains a copy of the Microarray Centre Quality Assessment of Affymetrix Data for EXP_CWTA_0103_01 comprising 138 hybridizations on Rat Expression Set 230A Arrays. Hybridization HFB2003080611Aaa listed in the QA report was excluded from the PCA dataset as the full annotation information was not available at the time of this study.

Microarray Centre Quality Assessment of Affymetrix Data

The quality assessment metrics described in this report are based on CEL intensity data from GeneChip® Affymetrix Arrays generated in the CSC/IC Microarray Centre using your samples.

The BioConductor (BioC Release 1.9) open source software was used to obtain the summary statistics reported (1).

NB. These procedures do not aim to provide guidelines to assess the overall quality of arrays but to aid the investigator in identifying outlier arrays within the set processed that can then be flagged and adjusted or excluded from further analysis. It is for this reason that specific thresholds are not provided as these need to be developed in the context of particular applications with specific types of samples.

Example data and a more detailed review of the statistical processes involved can be found on the CSC/IC Microarray Centre website (http://microarray.csc.mrc.ac.uk).

Further help with microarray data analysis can be obtained from:
Bioinformatics Support Service: bbshelp@ic.ac.uk
Statistical Advisory Service: stathelp@imperial.ac.uk

R Version: R version 2.5.0 (2007-04-23)

ExperimentID EXP_CWTA_0103_01

User Name: Caroline Wallace

Supervisor Name: Tim Aitman

Array Type: Rat Expression Set 230A Array

http://www.affymetrix.com/support/technical/byproduct.affx?product=rae230

Hybridisation ID	Sample ID
FFR2004022301Aaa	BN-Lx-6_f
FFR2004022302Aaa	SHR/Ola-8_f
FFR2004022303Aaa	RI 01-5_f
FFR2004022304Aaa	RI 02-3_f2
FFR2004022306Aaa	RI 05-4_f
FFR2004022308Aaa	RI 05c-5_f
FFR2004022309Aaa	RI 06c-4_f
FFR2004022310Aaa	RI 09c-5_f
FFR2004022501Aaa	RI 12c-5_f
FFR2004022502Aaa	RI 17-2_f
FFR2004022503Aaa	RI 03c-4_f
FFR2004022504Aaa	RI 13c-3_f
FFR2004022505Aaa	RI 15-6_f
FFR2004022506Aaa	RI 24-5_f
FFR2004022507Aaa	RI 25-3_f
FFR2004022508Aaa	RI 25-4_f
HFB2003050701Aaa	SHR/Ola-1_f
HFB2003050702Aaa	SHR/Ola-2_f
HFB2003050703Aaa	BN-Lx-1_f
HFB2003050705Aaa	RI 01-1_f
HFB2003050706Aaa	RI 02-2_f
HFB2003050707Aaa	RI 2c-1_f
HFB2003050708Aaa	RI 03-1_f

HFB2003050709Aaa	RI 05c-2_f
HFB2003050710Aaa	RI 06c-1_f
HFB2003050711Aaa	RI 07-1_f
HFB2003050712Aaa	RI 07-2_f
HFB2003050713Aaa	RI 09c-2_f
HFB2003050714Aaa	RI 10-2_f
HFB2003050715Aaa	RI 10c-2_f
HFB2003073101Aaa	RI 05-2_f
HFB2003073102Aaa	RI 04-2_f
HFB2003073103Aaa	RI 04-1_f
HFB2003073104Aaa	RI 11c-2_f
HFB2003073105Aaa	RI 02-1_f
HFB2003073106Aaa	RI 13c-2_f
HFB2003073107Aaa	RI 09c-1_f
HFB2003073108Aaa	RI 03-2_f
HFB2003073109Aaa	RI 03c-1_f
HFB2003073110Aaa	RI 03c-2_f
HFB2003073111Aaa	RI 11c-1_f
HFB2003080401Aaa	RI 18-1_f
HFB2003080402Aaa	RI 23-1_f
HFB2003080403Aaa	RI 20-2_f
HFB2003080404Aaa	RI 17-1_f
HFB2003080406Aaa	RI 21-2_f
HFB2003080407Aaa	RI 15-1_f
HFB2003080408Aaa	RI 08c-2_f
HFB2003080409Aaa	RI 15-2_f
HFB2003080410Aaa	RI 12c-2_f
HFB2003080411Aaa	RI 20-1_f
HFB2003080412Aaa	RI 18-2_f
HFB2003080601Aaa	RI 10c-1_f
HFB2003080602Aaa	RI 24-2_f
HFB2003080603Aaa	RI 23-2_f
HFB2003080604Aaa	RI 31-2_f
HFB2003080605Aaa	RI 22-2_f
HFB2003080606Aaa	RI 27-1_f
HFB2003080607Aaa	RI 13c-1_f
HFB2003080608Aaa	RI 26-2_f
HFB2003080609Aaa	RI 26-1_f
HFB2003080610Aaa	RI 31-1_f
HFB2003080611Aaa	RI 27-2_f
HFB2003080612Aaa	RI 29-2_f
HFB2003080613Aaa	RI 01-2_f
HFB2003080614Aaa	RI 29-1_f
NNC2003070701Aaa	BN-Lx-3_f
NNC2003070702Aaa	RI 01-4_f
NNC2003070703Aaa	RI 02-4_f
NNC2003070704Aaa	RI 2c-3_f
NNC2003070705Aaa	RI 2c-4_f
NNC2003070706Aaa	RI 03-4_f
NNC2003070707Aaa	RI 08c-3_f
NNC2003070708Aaa	RI 08c-4_f
NNC2003070709Aaa	RI 10-3_f
NNC2003070710Aaa	RI 10c-3_f
NNC2003070711Aaa	RI 11c-3_f
NNC2003070712Aaa	RI 11c-4_f
NNC2003070713Aaa	RI 12c-3_f
NNC2003070714Aaa	RI 12c-4_f
NNC2003092901Aaa	RI 02-3_f
NNC2003092903Aaa	RI 04-3_f
NNC2003092904Aaa	RI 04-4_f

NNC2003092905Aaa	RI 05-3_f
NNC2003092906Aaa	RI 05c-3_f
NNC2003092907Aaa	RI 07-3_f
NNC2003092908Aaa	RI 07-4_f
NNC2003092909Aaa	RI 13c-4_f
NNC2003092910Aaa	RI 03c-3_f
NNC2003100701Aaa	RI 17-3_f
NNC2003100702Aaa	RI 18-3_f
NNC2003100703Aaa	RI 18-4_f
NNC2003100705Aaa	RI 21-3_f
NNC2003100706Aaa	RI 22-3_f
NNC2003100707Aaa	RI 22-4_f
NNC2003100708Aaa	RI 23-3_f
NNC2003100710Aaa	RI 26-3_f
NNC2003100711Aaa	RI 27-4_f
NNC2003100712Aaa	RI 20-3_f
NNC2003100901Aaa	SHR/Ola-7_f
NNC2003100902Aaa	RI 08c-4_f
NNC2003100904Aaa	RI 24-3_f
NNC2003100905Aaa	RI 31-3_f
NNC2003100906Aaa	RI 31-4_f
NNC2003100907Aaa	RI 06c-6_f
NNC2003100909Aaa	BN-Lx-5_f
NNC2003102101Aaa	RI 02-3_f
NNC2003102102Aaa	RI 23-4_f
NNC2003102103Aaa	RI 03-3_f
NNC2003102104Aaa	RI 29-4_f
NNC2003102105Aaa	RI 12c-2_f
NNC2003102106Aaa	RI 24-2_f
NNC2003102107Aaa	RI 27-1_f
NNC2003102108Aaa	RI 31-1_f
NNC2003102109Aaa	RI 25-1_f
NNC2003102110Aaa	RI 27-2_f
NNC2003102111Aaa	RI 05c-1_f
NNC2003102112Aaa	RI 05-1_f
NNC2004020501Aaa	RI 06c-5_f
NNC2004020502Aaa	RI 2c-5_f
NNC2004020503Aaa	BN-Lx-2_f
NNC2004020504Aaa	RI 08c-5_f
NNC2004020507Aaa	RI 10-5_f
NNC2004020508Aaa	RI 25-5_f
NNC2004020509Aaa	RI 24-1_f
NNC2004020510Aaa	SHR/Ola-6_f
NNC2004020511Aaa	RI 22-1_f
NNC2004020512Aaa	RI 21-1_f
NNC2004022401Aaa	RI 09c-4_f
NNC2004022402Aaa	RI 10-4_f
NNC2004022403Aaa	RI 10c-5_f
NNC2004022404Aaa	RI 15-5_f
NNC2004022405Aaa	RI 17-4_f
NNC2004022406Aaa	RI 20-4_f
NNC2004022407Aaa	RI 21-4_f
NNC2004022408Aaa	RI 26-4_f
NNC2004022409Aaa	RI 27-3_f
NNC2004022410Aaa	RI 29-5_f

Exploratory visualisation is essential in detecting quality problems. The diagnostic plots described below are to aid this process and were generated using the affy package in BioConductor.

1. Probe-Intensity Distributions

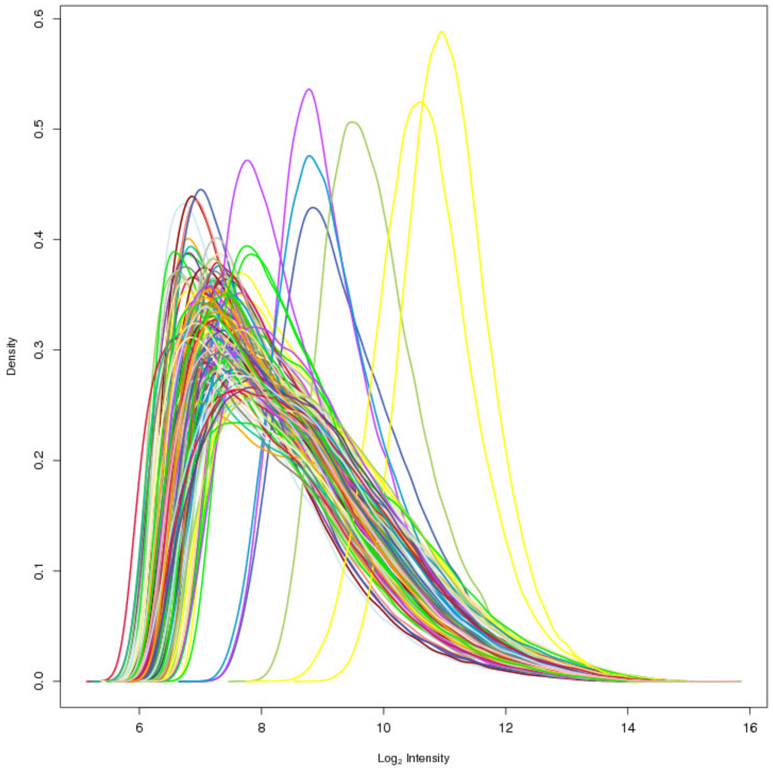

Smoothed histogram of the log2 probe intensities for each array before (i.e. unprocessed data) normalisation. These should be positively skewed (long right tail) and similar to each other in a sample set. If an array has a bimodal distribution this is usually indicative of a spatial artefact, the second mode being the result of an entire section of the array having abnormally high values. 'Blips' occuring at the high end of the log intensity scale could also indicate saturation of probes.

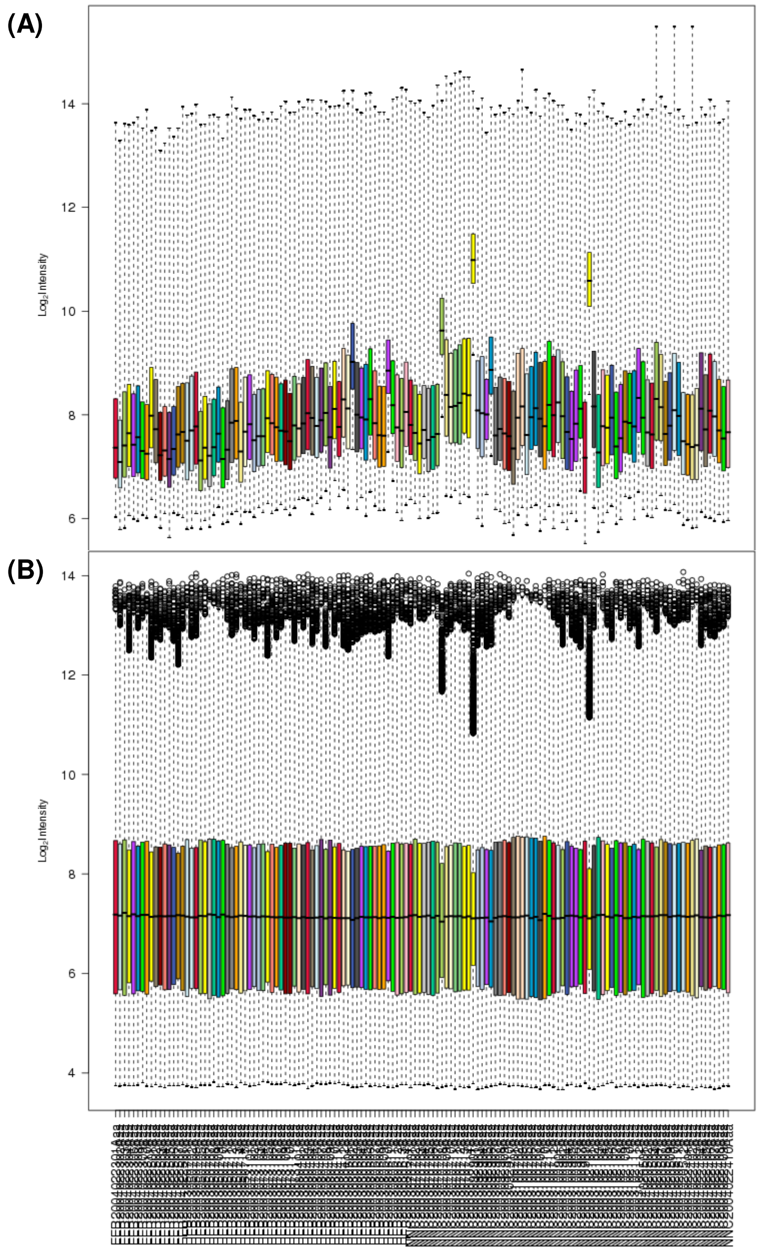

Box plots of the PM log2 probe intensities for each array. (A) Raw data before normalisation and (B) after RMA normalisation. The top and bottom sides of the box indicate the 75th and 25th percentiles of the log2 intensity distribution while the line within is the median. In general the intensity distributions should be similar across arrays. However, this does not mean that an array with an inconsistent distribution should be removed from analysis as normalisation can bring back the aberrant array into line with the other arrays. Good overlap of the box plots after normalisation indicates that there is not large variation in the log2 intensity values between each array.

2. RNA Degradation Plot

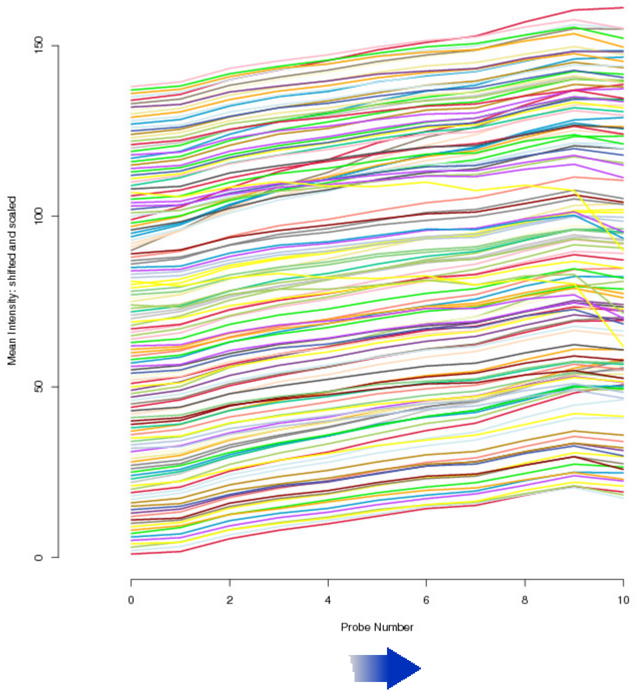

This plot shows the log2 mean intensity by probe pair position for each array. The probe pair position is obtained by sequentially numbering each individual probe of every probeset from the 5' end of the transcript to the 3' end. Probe intensities are then averaged by probe number across all genes on each array. Generally samples from a dataset should have similar shape slopes.

1. Relative Log2 Expression (RLE) Plots

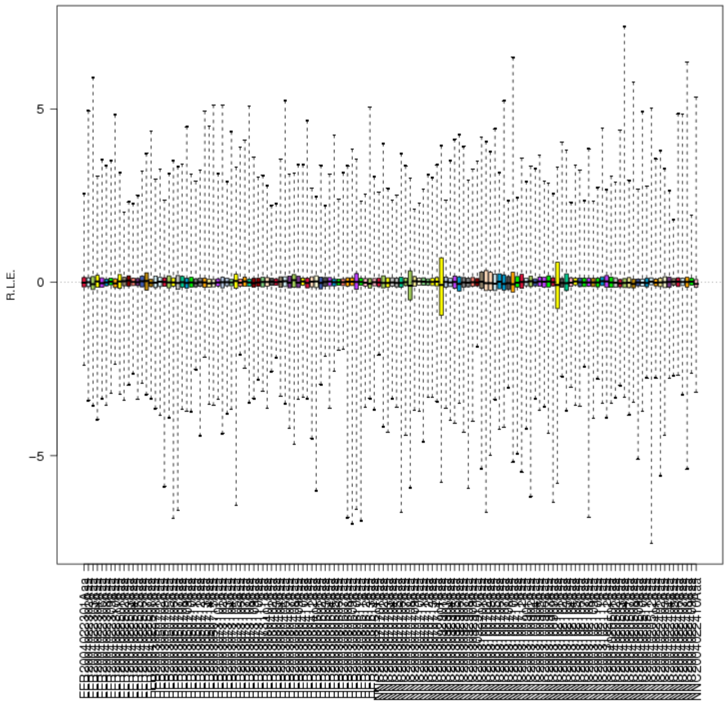

RLE is the log2 scale estimate of expression on each array relative to the median value across arrays on a probe set by probe set basis. Each box represents one array. Ideally these boxes would have small spread and be centred at 0. An array that has quality problems may result in a box that has greater spread or is not centred near 0.

2. Normalised Unscaled Standard Error (NUSE) Plots

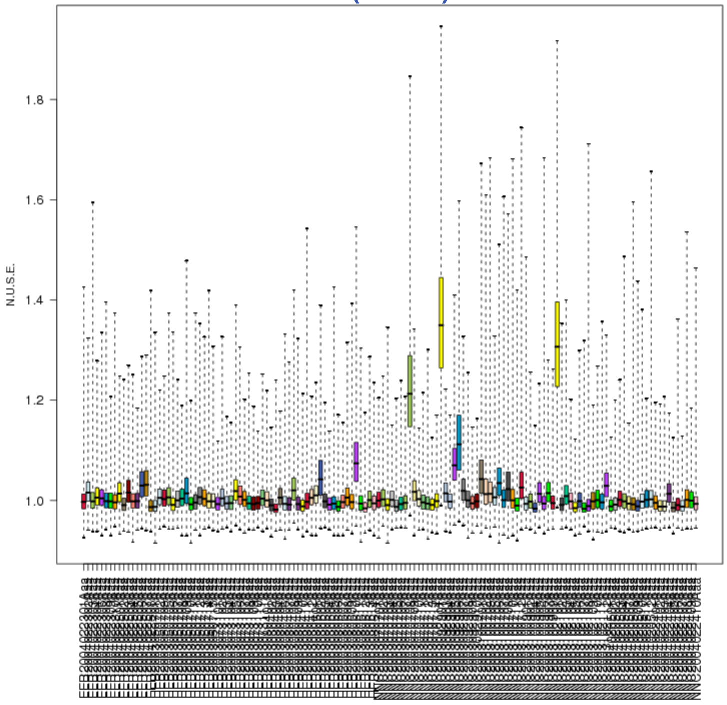

The goal of NUSE is to identify any arrays which have elevated standard errors relative to other arrays in the data set. This is done by standardising the SE across arrays to have a median of 1 for each probe set.

Affymetrix has developed several controls which allow researchers to monitor assay data quality (3). The following are a series of quality control parameters associated with assay and hybridisation performance. This data analysis was performed using the BioConductor statistical algorithms equating to those implemented in Affymetrix® Microarray Suite Version 5.0 (MAS 5.0) (for more information visit the IC/MaC or the Affymetrix websites).

ChipID	Average Background	Background StDev	Average Signal	%P	Scale Factor	RawQ
FFR2004022301Aaa	73.748	1.29	259.09	65.421	0.516	2.73
FFR2004022302Aaa	65.512	1.20	165.98	60.956	0.832	2.64
FFR2004022303Aaa	71.854	1.06	273.32	66.005	0.48	2.74
FFR2004022304Aaa	79.035	2.17	236.01	57.094	0.569	2.85
FFR2004022306Aaa	71.883	1.68	270.56	65.572	0.486	2.88
FFR2004022308Aaa	72.669	2.03	284.20	62.168	0.475	2.76
FFR2004022309Aaa	74.508	1.50	242.15	61.753	0.563	2.78
FFR2004022310Aaa	73.458	1.36	263.93	64.027	0.527	2.78
FFR2004022501Aaa	99.002	2.45	245.33	55.486	0.529	**3.52**
FFR2004022502Aaa	80.721	2.02	252.25	59.58	0.515	**3.07**
FFR2004022503Aaa	70.995	1.12	157.24	56.911	0.858	2.68
FFR2004022504Aaa	77.234	1.27	193.14	60.403	0.699	2.86
FFR2004022505Aaa	62.951	0.88	193.53	61.678	0.708	2.56
FFR2004022506Aaa	77.222	1.54	168.19	57.131	0.815	2.88
FFR2004022507Aaa	78.029	1.48	197.13	53.533	0.685	2.89
FFR2004022508Aaa	86.826	2.21	288.71	62.482	0.467	**3.10**
HFB2003050701Aaa	64.822	1.40	358.77	65.126	0.366	2.52
HFB2003050702Aaa	70.035	1.16	294.84	59.75	0.445	2.70
HFB2003050703Aaa	78.019	1.57	305.96	60.824	0.425	2.85
HFB2003050705Aaa	62.964	0.95	216.12	63.305	0.614	2.61
HFB2003050706Aaa	71.865	1.15	249.80	62.652	0.528	2.71
HFB2003050707Aaa	68.453	1.23	266.29	65.741	0.507	2.54
HFB2003050708Aaa	80.149	3.60	283.02	64.253	0.474	2.91
HFB2003050709Aaa	94.199	12.69	291.40	64.435	0.451	**3.17**
HFB2003050710Aaa	66.829	1.16	224.98	64.353	0.586	2.55
HFB2003050711Aaa	73.942	1.87	237.78	62.689	0.572	2.76
HFB2003050712Aaa	80.931	2.62	321.65	60.177	0.406	2.79
HFB2003050713Aaa	84.228	1.73	325.84	61.735	0.395	2.94
HFB2003050714Aaa	69.484	1.84	236.98	63.204	0.562	2.66
HFB2003050715Aaa	80.672	1.58	308.60	62.765	0.423	2.78
HFB2003073101Aaa	82.592	1.49	299.72	62.149	0.435	**3.31**
HFB2003073102Aaa	74.766	1.99	218.79	60.548	0.623	**3.10**
HFB2003073103Aaa	81.884	1.64	253.06	61.157	0.529	**3.17**
HFB2003073104Aaa	84.028	2.98	267.51	61.496	0.506	**3.29**
HFB2003073105Aaa	94.898	2.72	247.92	55.291	0.537	**3.42**
HFB2003073106Aaa	93.515	8.05	302.36	62.099	0.431	**3.51**
HFB2003073107Aaa	84.827	2.78	270.35	58.5	0.491	**3.26**
HFB2003073108Aaa	90.806	2.12	283.30	61.044	0.476	**3.48**
HFB2003073109Aaa	83.083	1.95	330.43	62.84	0.406	**3.20**
HFB2003073110Aaa	81.497	2.10	272.61	62.664	0.503	**3.14**

HFB2003073111Aaa	85.401	2.23	278.39	58.437	0.482	**3.27**
HFB2003080401Aaa	96.654	2.34	295.97	60.234	0.461	**3.96**
HFB2003080402Aaa	99.479	2.04	301.72	60.152	0.448	**3.81**
HFB2003080403Aaa	97.241	1.79	407.66	63.072	0.32	**3.65**
HFB2003080404Aaa	87.168	1.65	326.54	58.456	0.414	**3.40**
HFB2003080406Aaa	92.655	1.95	285.32	59.951	0.468	**3.54**
HFB2003080407Aaa	97.873	3.60	392.23	64.184	0.335	**3.79**
HFB2003080408Aaa	**106.907**	2.04	303.80	55.272	0.442	**3.93**
HFB2003080409Aaa	82.617	2.41	311.64	63.336	0.434	**3.23**
HFB2003080410Aaa	**122.792**	3.83	343.75	59.292	0.389	**4.41**
HFB2003080411Aaa	98.241	2.83	311.63	62.501	0.434	**3.73**
HFB2003080412Aaa	**110.685**	3.18	404.92	57.571	0.325	**3.97**
HFB2003080601Aaa	88.2	2.21	342.55	57.018	0.383	**3.37**
HFB2003080602Aaa	**197.195**	6.63	453.57	58.437	0.285	**6.03**
HFB2003080603Aaa	90.315	1.88	326.81	59.348	0.403	**3.38**
HFB2003080604Aaa	97.963	2.64	298.22	59.618	0.443	**3.62**
HFB2003080605Aaa	77.96	1.91	370.47	61.898	0.361	2.97
HFB2003080606Aaa	**113.283**	2.94	430.40	62.306	0.303	**4.04**
HFB2003080607Aaa	85.764	2.93	326.53	62.03	0.408	**3.19**
HFB2003080608Aaa	82.327	2.28	269.33	60.523	0.507	**3.23**
HFB2003080609Aaa	81.906	1.42	267.72	60.837	0.507	**3.16**
HFB2003080610Aaa	**198.548**	8.66	292.09	53.809	0.455	**6.04**
HFB2003080611Aaa	**125.246**	3.09	359.56	62.363	0.37	**4.36**
HFB2003080612Aaa	84.756	1.84	354.85	63.675	0.378	**3.19**
HFB2003080613Aaa	74.312	1.85	329.46	64.536	0.407	3.00
HFB2003080614Aaa	**101.273**	4.74	390.58	63.864	0.335	**3.99**
NNC2003070701Aaa	96.84	2.23	287.93	62.721	0.465	**3.75**
NNC2003070702Aaa	92.959	1.58	308.09	64.039	0.43	**3.62**
NNC2003070703Aaa	76.99	1.52	303.18	63.813	0.462	2.97
NNC2003070704Aaa	97.066	2.25	286.71	61.747	0.479	**3.70**
NNC2003070705Aaa	77.654	2.03	296.69	65.233	0.447	2.99
NNC2003070706Aaa	78.796	1.56	338.97	63.606	0.408	**3.27**
NNC2003070707Aaa	83.103	2.32	340.19	63.38	0.41	**3.54**
NNC2003070708Aaa	**327.074**	15.49	327.06	47.673	0.399	**7.79**
NNC2003070709Aaa	**106.071**	3.85	464.93	59.781	0.28	**4.12**
NNC2003070710Aaa	**106.316**	4.16	447.31	61.301	0.302	**4.18**
NNC2003070711Aaa	**105.141**	3.76	504.80	62.457	0.265	**4.08**
NNC2003070712Aaa	93.678	3.87	519.19	63.11	0.253	**3.85**
NNC2003070713Aaa	**110.074**	3.73	521.84	61.584	0.252	**4.33**
NNC2003070714Aaa	**104.466**	2.55	519.70	62.218	0.249	**4.05**
NNC2003092901Aaa	**791.239**	69.61	306.73	32.042	0.4	**15.16**
NNC2003092903Aaa	87.5	2.27	292.91	57.458	0.441	**3.33**
NNC2003092904Aaa	73.542	2.49	394.62	61.98	0.329	2.89
NNC2003092905Aaa	**111.915**	3.70	193.01	54.481	0.691	**4.25**
NNC2003092906Aaa	**187.748**	9.30	341.38	58.632	0.383	**6.13**
NNC2003092907Aaa	85.14	4.81	285.28	62.557	0.459	**3.28**
NNC2003092908Aaa	87.806	4.69	341.95	64.661	0.382	**3.34**
NNC2003092909Aaa	74.53	2.10	343.80	64.473	0.379	**3.08**

NNC2003092910Aaa	74.062	1.58	301.71	62.243	0.44	**3.02**
NNC2003100701Aaa	60.849	0.99	308.09	63.116	0.427	2.53
NNC2003100702Aaa	79.566	1.62	556.93	64.868	0.225	**3.15**
NNC2003100703Aaa	94.237	1.96	576.57	64.542	0.217	**3.54**
NNC2003100705Aaa	66.268	1.56	416.30	66.495	0.305	2.69
NNC2003100706Aaa	94.221	2.14	390.82	62.62	0.33	**3.42**
NNC2003100707Aaa	98.133	3.07	503.67	64.894	0.25	**3.47**
NNC2003100708Aaa	82.226	16.85	411.84	63.845	0.306	2.93
NNC2003100710Aaa	72.018	1.51	498.68	67.041	0.253	2.71
NNC2003100711Aaa	85.979	2.19	564.71	65.591	0.221	**3.16**
NNC2003100712Aaa	77.491	5.90	440.73	62.18	0.293	2.91
NNC2003100901Aaa	95.789	2.72	418.88	62.451	0.301	**3.75**
NNC2003100902Aaa	73.467	1.82	332.74	59.034	0.394	**3.07**
NNC2003100904Aaa	72.113	1.55	335.60	64.624	0.385	2.92
NNC2003100905Aaa	76.628	1.44	218.64	60.24	0.605	2.96
NNC2003100906Aaa	78.589	1.82	298.58	60.372	0.435	**3.08**
NNC2003100907Aaa	**113.416**	3.42	274.55	56.114	0.487	**3.95**
NNC2003100909Aaa	56.628	1.69	241.92	64.234	0.554	2.30
NNC2003102101Aaa	**516.767**	23.92	373.44	41.701	0.339	**11.46**
NNC2003102102Aaa	92.387	1.86	406.66	63.895	0.311	**3.55**
NNC2003102103Aaa	61.621	1.29	337.63	67.154	0.389	2.49
NNC2003102104Aaa	74.905	1.40	379.57	66.52	0.337	2.99
NNC2003102105Aaa	81.341	2.60	312.00	61.628	0.424	**3.15**
NNC2003102106Aaa	90.451	2.86	333.32	59.781	0.389	**3.32**
NNC2003102107Aaa	71.839	1.53	300.51	65.528	0.441	2.75
NNC2003102108Aaa	76.739	1.51	297.70	64.146	0.437	2.92
NNC2003102109Aaa	**100.963**	1.52	348.37	62.545	0.372	**3.65**
NNC2003102110Aaa	95.355	2.46	281.62	60.183	0.461	**3.43**
NNC2003102111Aaa	82.318	2.46	333.65	60.51	0.386	2.98
NNC2003102112Aaa	**119.459**	11.52	374.17	57.382	0.342	**3.88**
NNC2004020501Aaa	90.318	3.29	446.71	65.861	0.283	**3.58**
NNC2004020502Aaa	79.109	2.09	346.50	66.482	0.375	**3.14**
NNC2004020503Aaa	77.922	1.79	313.95	65.283	0.419	**3.23**
NNC2004020504Aaa	97.804	3.04	469.54	64.485	0.268	**3.84**
NNC2004020507Aaa	**111.22**	4.19	480.37	66.583	0.26	**4.15**
NNC2004020508Aaa	83.973	2.27	323.37	64.837	0.407	**3.30**
NNC2004020509Aaa	84.593	1.61	325.67	63.047	0.393	**3.34**
NNC2004020510Aaa	88.778	2.55	424.98	63.606	0.294	**3.34**
NNC2004020511Aaa	83.248	2.77	386.71	64.611	0.327	**3.20**
NNC2004020512Aaa	69.978	1.35	252.12	61.923	0.526	2.75
NNC2004022401Aaa	72.253	1.30	262.51	63.085	0.507	2.68
NNC2004022402Aaa	67.815	1.07	289.57	65.145	0.45	2.66
NNC2004022403Aaa	67.32	0.94	324.25	65.578	0.396	2.62
NNC2004022404Aaa	82.476	1.37	374.07	60.246	0.339	**3.07**
NNC2004022405Aaa	76.262	1.78	333.99	61.194	0.395	2.92
NNC2004022406Aaa	79.28	2.38	387.71	59.951	0.333	**3.03**
NNC2004022407Aaa	75.074	1.73	331.08	59.907	0.384	**3.01**
NNC2004022408Aaa	80.745	1.78	266.42	62.187	0.489	**3.10**

NNC2004022409Aaa	72.162	1.57	255.21	62.639	0.507	2.94
NNC2004022410Aaa	74.191	1.38	326.54	65.842	0.396	2.84

1. Average Background

Average Background values are a measure of the signal intensity caused by autofluoresence of the array surface as well as non-specific binding of target or SAPE stain molecules. It is calculated by taking the mean average signal value of the lowest 2% probes. Values should range from 20 to 100 for arrays scanned with the GeneChip® Scanner 3000. Arrays being compared should ideally have comparable background values.

2. Percent Present

Percent Present (%P) is a measure of the percent of genes on the array that are marked as being expressed (where the Perfect Match (PM) probe signal is seen as being significantly higher than the Mismatch (MM) probe signal). Expect to see %P to be ~50% for whole genome arrays and ~25% for Test3 arrays. Low %P values might indicate reduced RNA quality and are usually seen with high non-specific binding, (where the MM probe may be giving a signal value closer to PM probe signal value).

3. Scale Factors

Global scaling was applied to a Target Intensity of 100 across all arrays. For replicates and comparisons involving a relatively small number of changes, the scaling/normalization factors (calculated by the global method) should be comparable among arrays. Larger discrepancies among scaling/normalization factors (e.g., three-fold or greater) may indicate significant assay variability or sample degradation leading to noisier data.

4. RawQ Noise

Noise (Raw Q) is a measure of the pixel-to-pixel variation of probe cells on a GeneChip array. The two main factors that contribute to noise are electrical noise of the scanner and sample quality. Each scanner has a unique inherent electrical noise associated with its operation. Since a significant portion of Noise (Raw Q) is electrical noise, values among scanners will vary. Array data (especially those of replicates) acquired from the same scanner should ideally have comparable Noise values.

5. Hybridisation Controls

A mixture of biotin-labeled cRNA transcripts of bioB, bioC, bioD (genes in the biotin synthesis pathway of E.coli) and cre (recombinase gene from P1 bacteriophage), is prepared in staggered concentrations (1.5 pM, 5 pM, 25 pM, and 100 pM final concentrations for bioB, bioC, bioD, and cre, respectively). These are spiked into the hybridisation cocktail prior to hybridisation, and are thus used to evaluate sample hybridisation efficiency. bioB, bioC, bioD, and cre should always give increasing Signal values, reflecting their relative concentrations.

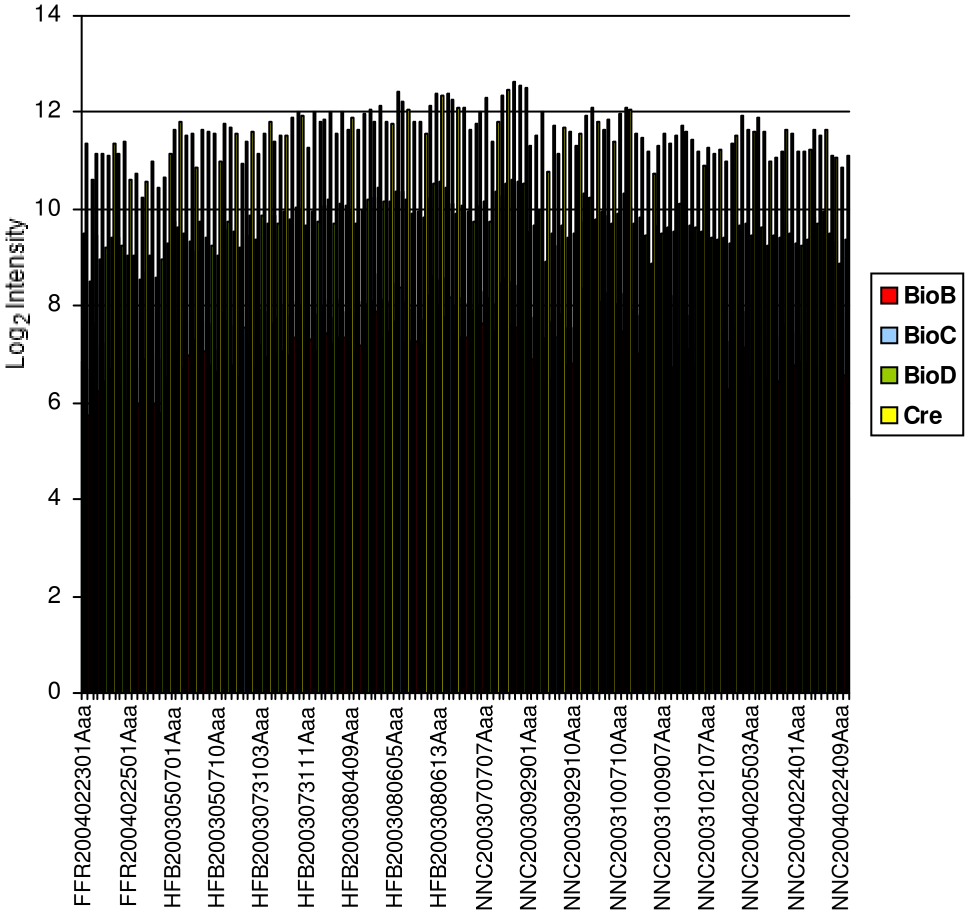

6. Poly A+ Controls

A pre-synthesized mixture of lys, phe, dap, thr and trp RNA controls (from specially modified B. subtilis genes) is spiked into each RNA sample in staggered concentrations. These are carried through the entire sample preparation process, and evaluated like internal control genes. All of the PolyA+ controls should be called "Present" with increasing signal values in the following order: trp>thr>dap>phe>lys and can be used to monitor the entire target labelling process.

Concentration (ng/µl)	Poly A+ Control
14.7	TrpX
39.1	ThrX
108.9	DapX
260.0	PheX
591.1	LysX

A bar graph is plotted to check for consistency of signal.
We would expect to see excellent correlation between their concentration and raw signal value (R2 greater than 0.95). Below are the R2 correlation values of log2 transcript concentration (ng/µl) vs. log2 signal (fluorescent units). Low correlation could indicate poor labelling and/or poor sample handling.

ChipID	R^2
FFR2004022301Aaa	0.9839
FFR2004022302Aaa	0.9855
FFR2004022303Aaa	0.9813
FFR2004022304Aaa	0.9888
FFR2004022306Aaa	0.9880
FFR2004022308Aaa	0.9885
FFR2004022309Aaa	0.9772
FFR2004022310Aaa	0.9758
FFR2004022501Aaa	0.9868
FFR2004022502Aaa	0.9881
FFR2004022503Aaa	0.9783
FFR2004022504Aaa	0.9723
FFR2004022505Aaa	0.9850
FFR2004022506Aaa	0.9860
FFR2004022507Aaa	0.9834
FFR2004022508Aaa	0.9813
HFB2003050701Aaa	0.9695
HFB2003050702Aaa	0.9702
HFB2003050703Aaa	0.9806
HFB2003050705Aaa	0.9609
HFB2003050706Aaa	0.9805
HFB2003050707Aaa	0.9698
HFB2003050708Aaa	0.9736
HFB2003050709Aaa	0.9775
HFB2003050710Aaa	0.9729
HFB2003050711Aaa	0.9815
HFB2003050712Aaa	0.9784
HFB2003050713Aaa	0.9781
HFB2003050714Aaa	0.9669
HFB2003050715Aaa	0.9801
HFB2003073101Aaa	0.9682
HFB2003073102Aaa	0.9702
HFB2003073103Aaa	0.9698
HFB2003073104Aaa	0.9736
HFB2003073105Aaa	0.9823
HFB2003073106Aaa	0.9737
HFB2003073107Aaa	0.9751
HFB2003073108Aaa	0.9692
HFB2003073109Aaa	0.9731
HFB2003073110Aaa	0.9689
HFB2003073111Aaa	0.9727
HFB2003080401Aaa	0.9733
HFB2003080402Aaa	0.9738
HFB2003080403Aaa	0.9675
HFB2003080404Aaa	0.9831
HFB2003080406Aaa	0.9743
HFB2003080407Aaa	0.9738
HFB2003080408Aaa	0.9751
HFB2003080409Aaa	0.9706
HFB2003080410Aaa	0.9742
HFB2003080411Aaa	0.9705
HFB2003080412Aaa	0.9813
HFB2003080601Aaa	0.9834
HFB2003080602Aaa	0.9778
HFB2003080603Aaa	0.9816
HFB2003080604Aaa	0.9851
HFB2003080605Aaa	0.9783
HFB2003080606Aaa	0.9808

HFB2003080607Aaa	0.9719
HFB2003080608Aaa	0.9764
HFB2003080609Aaa	0.9759
HFB2003080610Aaa	0.9751
HFB2003080611Aaa	0.9778
HFB2003080612Aaa	0.9730
HFB2003080613Aaa	0.9598
HFB2003080614Aaa	0.9720
NNC2003070701Aaa	0.9794
NNC2003070702Aaa	0.9757
NNC2003070703Aaa	0.9761
NNC2003070704Aaa	0.9793
NNC2003070705Aaa	0.9775
NNC2003070706Aaa	0.9731
NNC2003070707Aaa	0.9743
NNC2003070708Aaa	0.9777
NNC2003070709Aaa	0.9642
NNC2003070710Aaa	0.9776
NNC2003070711Aaa	0.9803
NNC2003070712Aaa	0.9745
NNC2003070713Aaa	0.9763
NNC2003070714Aaa	0.9765
NNC2003092901Aaa	0.9956
NNC2003092903Aaa	0.9808
NNC2003092904Aaa	0.9780
NNC2003092905Aaa	0.9804
NNC2003092906Aaa	0.9833
NNC2003092907Aaa	0.9745
NNC2003092908Aaa	0.9854
NNC2003092909Aaa	0.9749
NNC2003092910Aaa	0.9792
NNC2003100701Aaa	0.9751
NNC2003100702Aaa	0.9674
NNC2003100703Aaa	0.9751
NNC2003100705Aaa	0.9733
NNC2003100706Aaa	0.9638
NNC2003100707Aaa	0.9742
NNC2003100708Aaa	0.9781
NNC2003100710Aaa	0.9785
NNC2003100711Aaa	0.9818
NNC2003100712Aaa	0.9874
NNC2003100901Aaa	0.9746
NNC2003100902Aaa	0.9818
NNC2003100904Aaa	0.9764
NNC2003100905Aaa	0.9765
NNC2003100906Aaa	0.9823
NNC2003100907Aaa	0.9796
NNC2003100909Aaa	0.9755
NNC2003102101Aaa	0.9863
NNC2003102102Aaa	0.9821
NNC2003102103Aaa	0.9668
NNC2003102104Aaa	0.9825
NNC2003102105Aaa	0.9774
NNC2003102106Aaa	0.9828
NNC2003102107Aaa	0.9758
NNC2003102108Aaa	0.9654
NNC2003102109Aaa	0.9729
NNC2003102110Aaa	0.9791
NNC2003102111Aaa	0.9654
NNC2003102112Aaa	0.9816

NNC2004020501Aaa	0.9736
NNC2004020502Aaa	0.9686
NNC2004020503Aaa	0.9841
NNC2004020504Aaa	0.9858
NNC2004020507Aaa	0.9742
NNC2004020508Aaa	0.9650
NNC2004020509Aaa	0.9718
NNC2004020510Aaa	0.9816
NNC2004020511Aaa	0.9777
NNC2004020512Aaa	**0.4498**
NNC2004022401Aaa	0.9763
NNC2004022402Aaa	0.9718
NNC2004022403Aaa	0.9778
NNC2004022404Aaa	0.9848
NNC2004022405Aaa	0.9843
NNC2004022406Aaa	0.9779
NNC2004022407Aaa	0.9801
NNC2004022408Aaa	0.9768
NNC2004022409Aaa	0.9797
NNC2004022410Aaa	0.9838

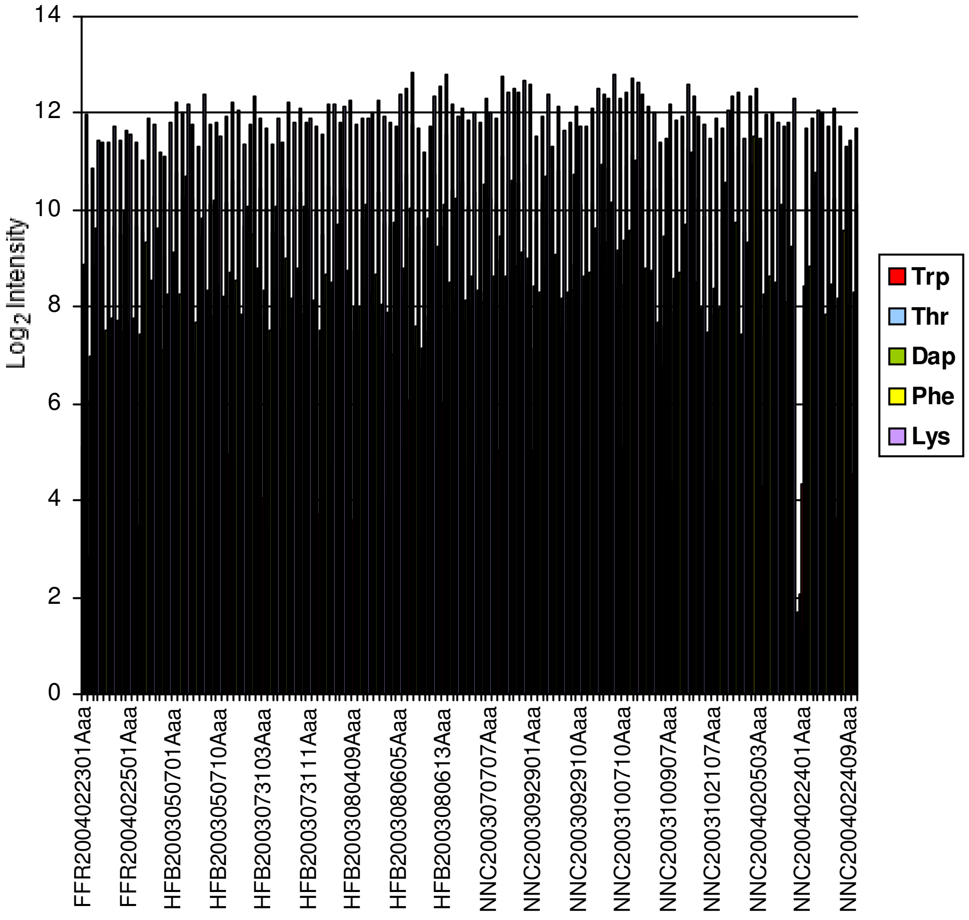

7. Housekeeping Genes

The housekeeping genes, GAPDH and β-actin, give an indication as to the quality of your samples. We expect these genes to be expressed in all cell types and therefore generate signal greater than 2500 fluorescent units for every sample. The signals should generally be consistent across the data set, although these two genes can sometimes vary between factor groups (eg treated samples vs. untreated samples), but should be consistent within factor groups. There may also be more variation between clinical samples as opposed to cells grown in culture.

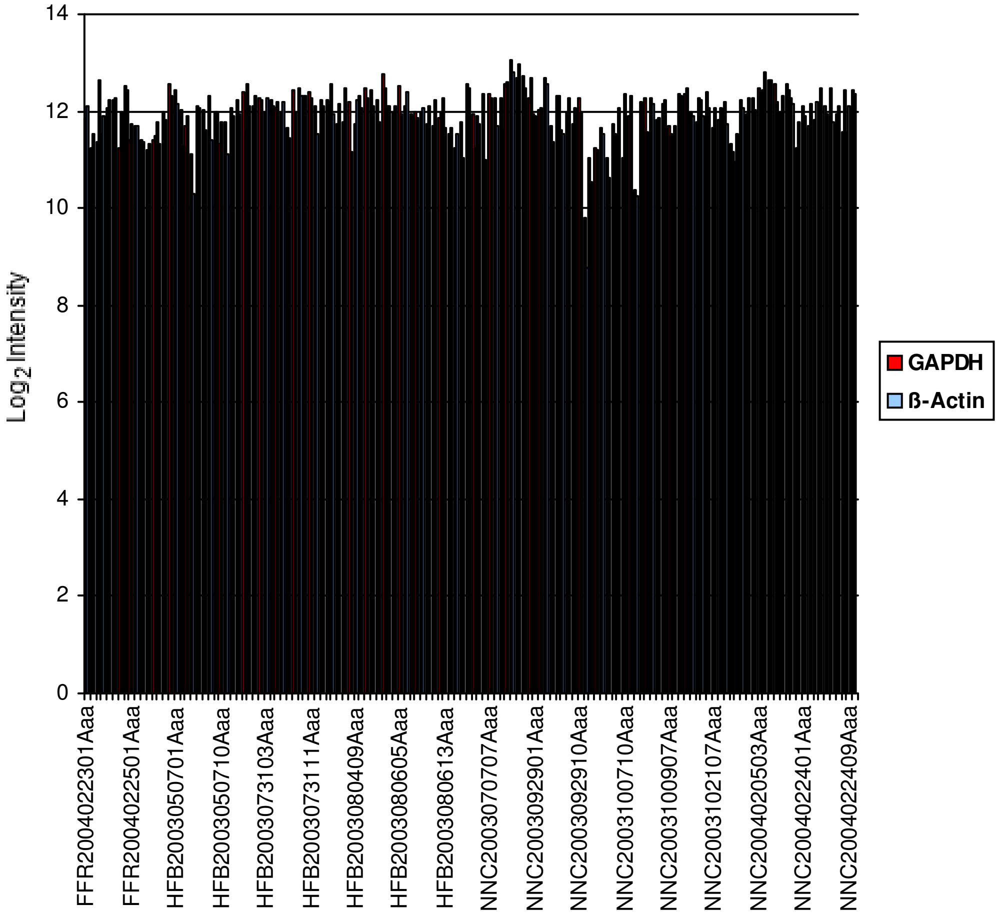

8. 3'/5' Ratios

The Affymetrix arrays contain probesets designed to 3' and 5' regions of the PolyA+ controls and housekeeping gene transcripts. Since the Affymetrix eukaryotic expression assay has an inherent 3' bias, a high 3' to 5' ratio may indicate degraded RNA or inefficient transcription of cDNA or biotinylated cRNA. The small sample labelling assay typically gives higher 3' to 5' ratios than the standard assay, due to the additional cycle of amplification.
There are occasions when the 3' to 5' ratio of one internal control gene is normal, but the 3' to 5' ratio of another internal control gene is high. This discrepancy in 3' to 5' ratios is most likely due to a specific transcript-related or image artifact issue and is not an indication of overall sample and assay quality.
Affymetrix suggest that 3'/5' ratios below 3 indicate sufficient labeling efficiency and good sample quality for the standard, one cycle protocol. This threshold is less important for the small sample, (two cycle) labelling assay.

CEL FILE	Phe	Lys	GAPDH	B-Actin
FFR2004022301Aaa	**3.07**	1.70	1.32	1.33
FFR2004022302Aaa	**5.48**	**3.02**	1.73	1.63
FFR2004022303Aaa	**3.44**	2.26	1.51	1.23
FFR2004022304Aaa	**3.20**	2.42	1.46	1.56
FFR2004022306Aaa	**3.97**	2.51	1.26	1.13
FFR2004022308Aaa	**3.53**	2.23	1.31	1.43
FFR2004022309Aaa	**4.61**	2.38	2.08	1.52
FFR2004022310Aaa	**3.63**	2.15	1.40	1.20
FFR2004022501Aaa	**3.69**	2.35	1.67	1.63
FFR2004022502Aaa	**3.41**	2.37	1.46	1.60
FFR2004022503Aaa	**3.98**	2.54	1.66	1.41
FFR2004022504Aaa	**3.10**	1.50	1.49	1.71
FFR2004022505Aaa	**3.64**	1.95	1.67	2.01
FFR2004022506Aaa	**3.79**	2.74	1.50	2.73
FFR2004022507Aaa	**3.86**	2.81	1.29	1.38
FFR2004022508Aaa	**3.35**	2.23	1.25	1.35
HFB2003050701Aaa	2.92	1.65	1.16	1.54
HFB2003050702Aaa	2.27	2.22	1.35	2.40
HFB2003050703Aaa	2.37	1.83	1.25	1.94
HFB2003050705Aaa	2.73	1.85	2.22	**3.40**
HFB2003050706Aaa	2.97	2.62	1.27	1.78
HFB2003050707Aaa	2.64	1.48	1.49	2.62
HFB2003050708Aaa	**3.97**	2.46	1.43	2.43
HFB2003050709Aaa	**3.01**	2.20	1.39	1.55
HFB2003050710Aaa	2.97	2.08	1.54	1.70
HFB2003050711Aaa	2.85	2.36	1.54	**3.15**
HFB2003050712Aaa	2.30	1.98	1.20	1.90
HFB2003050713Aaa	2.24	1.96	1.23	1.45
HFB2003050714Aaa	2.90	2.52	1.15	1.66
HFB2003050715Aaa	2.38	2.07	1.17	1.22
HFB2003073101Aaa	2.65	1.51	1.33	1.41
HFB2003073102Aaa	2.72	1.55	1.16	1.06
HFB2003073103Aaa	2.92	1.84	1.32	1.20
HFB2003073104Aaa	2.89	2.24	1.31	1.32
HFB2003073105Aaa	2.41	1.94	1.13	1.42
HFB2003073106Aaa	2.57	2.54	1.32	1.41
HFB2003073107Aaa	2.50	1.71	1.93	2.15
HFB2003073108Aaa	2.59	2.16	1.25	1.23
HFB2003073109Aaa	2.72	1.83	1.34	1.53
HFB2003073110Aaa	2.78	1.86	1.34	1.31
HFB2003073111Aaa	2.28	1.84	1.26	1.24
HFB2003080401Aaa	**3.52**	2.33	1.50	2.78
HFB2003080402Aaa	2.69	2.35	1.45	1.37
HFB2003080403Aaa	2.44	1.83	1.49	1.70
HFB2003080404Aaa	2.10	1.88	1.24	1.90
HFB2003080406Aaa	2.66	2.24	1.22	1.66

HFB2003080407Aaa	**3.19**	1.89	1.75	1.64
HFB2003080408Aaa	2.92	2.08	1.59	2.27
HFB2003080409Aaa	**3.79**	2.25	1.69	1.73
HFB2003080410Aaa	2.67	2.15	1.41	1.70
HFB2003080411Aaa	2.69	2.23	1.34	1.32
HFB2003080412Aaa	2.52	2.35	1.30	1.68
HFB2003080601Aaa	2.39	2.02	1.29	1.84
HFB2003080602Aaa	2.69	2.42	1.26	1.40
HFB2003080603Aaa	2.08	2.21	1.25	1.85
HFB2003080604Aaa	2.23	2.34	1.15	1.54
HFB2003080605Aaa	2.10	1.96	1.37	1.85
HFB2003080606Aaa	2.06	1.82	1.27	1.60
HFB2003080607Aaa	1.82	1.21	1.43	1.82
HFB2003080608Aaa	2.28	2.66	1.41	2.04
HFB2003080609Aaa	2.94	2.62	1.41	1.72
HFB2003080610Aaa	2.32	2.00	1.24	1.70
HFB2003080611Aaa	2.54	1.60	1.61	1.58
HFB2003080612Aaa	2.24	1.61	1.53	2.22
HFB2003080613Aaa	2.05	1.32	1.26	1.67
HFB2003080614Aaa	2.61	2.13	1.78	2.09
NNC2003070701Aaa	2.82	2.14	1.69	2.39
NNC2003070702Aaa	2.78	2.21	1.75	2.10
NNC2003070703Aaa	**3.27**	2.35	1.19	1.36
NNC2003070704Aaa	**3.06**	2.16	1.80	2.47
NNC2003070705Aaa	2.51	1.92	1.55	1.72
NNC2003070706Aaa	**3.74**	2.11	1.38	**3.16**
NNC2003070707Aaa	**4.01**	2.93	1.64	1.82
NNC2003070708Aaa	2.90	2.35	1.32	1.94
NNC2003070709Aaa	**3.66**	2.20	2.02	2.82
NNC2003070710Aaa	**3.84**	2.50	1.54	1.94
NNC2003070711Aaa	2.70	2.11	1.32	1.83
NNC2003070712Aaa	2.90	2.30	1.33	1.59
NNC2003070713Aaa	**3.31**	2.12	1.52	1.79
NNC2003070714Aaa	2.65	2.10	1.43	1.71
NNC2003092901Aaa	1.93	2.23	1.32	1.87
NNC2003092903Aaa	2.74	2.27	1.11	1.67
NNC2003092904Aaa	2.31	1.95	1.09	1.47
NNC2003092905Aaa	**3.62**	1.95	1.51	1.86
NNC2003092906Aaa	2.61	2.14	1.35	1.28
NNC2003092907Aaa	**4.29**	2.30	2.23	2.05
NNC2003092908Aaa	2.85	2.56	1.37	1.59
NNC2003092909Aaa	2.90	1.70	2.11	2.06
NNC2003092910Aaa	**3.50**	2.71	1.26	1.74
NNC2003100701Aaa	**30.32**	**3.45**	**62.50**	**12.76**
NNC2003100702Aaa	**18.91**	**3.87**	**12.63**	**4.81**
NNC2003100703Aaa	**11.10**	2.08	**25.85**	**4.89**
NNC2003100705Aaa	**5.42**	2.01	**3.84**	2.64
NNC2003100706Aaa	**6.82**	2.29	**8.98**	**4.75**
NNC2003100707Aaa	**3.15**	1.54	**7.47**	**4.25**
NNC2003100708Aaa	**3.20**	1.79	**5.26**	2.12
NNC2003100710Aaa	**3.99**	1.63	**15.85**	**3.16**
NNC2003100711Aaa	2.64	1.70	1.99	2.03
NNC2003100712Aaa	**6.70**	1.91	**33.21**	**6.24**
NNC2003100901Aaa	**3.28**	1.86	2.13	2.52
NNC2003100902Aaa	2.50	2.01	1.23	1.77
NNC2003100904Aaa	2.91	1.96	1.31	1.39
NNC2003100905Aaa	2.99	2.09	1.29	1.55
NNC2003100906Aaa	2.68	2.52	1.09	1.32
NNC2003100907Aaa	2.90	1.83	1.77	2.05

NNC2003100909Aaa	**3.22**	1.97	1.78	2.03
NNC2003102101Aaa	2.26	2.31	1.27	1.49
NNC2003102102Aaa	2.21	1.50	1.22	1.39
NNC2003102103Aaa	2.81	1.64	2.69	1.78
NNC2003102104Aaa	2.75	1.97	1.46	1.73
NNC2003102105Aaa	**3.30**	2.20	1.37	1.80
NNC2003102106Aaa	**3.35**	2.50	1.24	1.38
NNC2003102107Aaa	2.70	2.03	1.35	1.52
NNC2003102108Aaa	2.12	2.01	1.34	1.41
NNC2003102109Aaa	**3.14**	1.91	1.57	2.43
NNC2003102110Aaa	2.74	1.49	1.86	2.18
NNC2003102111Aaa	2.79	1.65	**4.73**	2.62
NNC2003102112Aaa	2.64	2.59	1.35	1.79
NNC2004020501Aaa	**3.14**	1.66	1.52	1.95
NNC2004020502Aaa	2.48	1.36	1.23	1.62
NNC2004020503Aaa	2.90	2.56	1.20	1.26
NNC2004020504Aaa	2.31	2.20	1.15	1.29
NNC2004020507Aaa	2.68	2.00	1.24	1.38
NNC2004020508Aaa	2.95	2.15	1.22	1.38
NNC2004020509Aaa	**3.07**	2.30	1.25	1.35
NNC2004020510Aaa	2.77	2.12	1.23	1.29
NNC2004020511Aaa	2.65	1.59	1.28	1.53
NNC2004020512Aaa	1.52	2.28	1.27	1.83
NNC2004022401Aaa	**4.36**	2.14	1.40	1.46
NNC2004022402Aaa	**4.07**	2.04	1.48	1.41
NNC2004022403Aaa	**3.23**	1.68	1.48	1.45
NNC2004022404Aaa	2.87	2.08	1.26	1.30
NNC2004022405Aaa	**4.32**	2.45	1.37	1.74
NNC2004022406Aaa	**3.94**	2.28	1.37	1.97
NNC2004022407Aaa	**4.04**	2.53	1.29	1.46
NNC2004022408Aaa	**4.30**	2.58	1.56	1.21
NNC2004022409Aaa	**4.17**	2.22	1.58	1.39
NNC2004022410Aaa	**4.31**	2.31	1.29	1.27

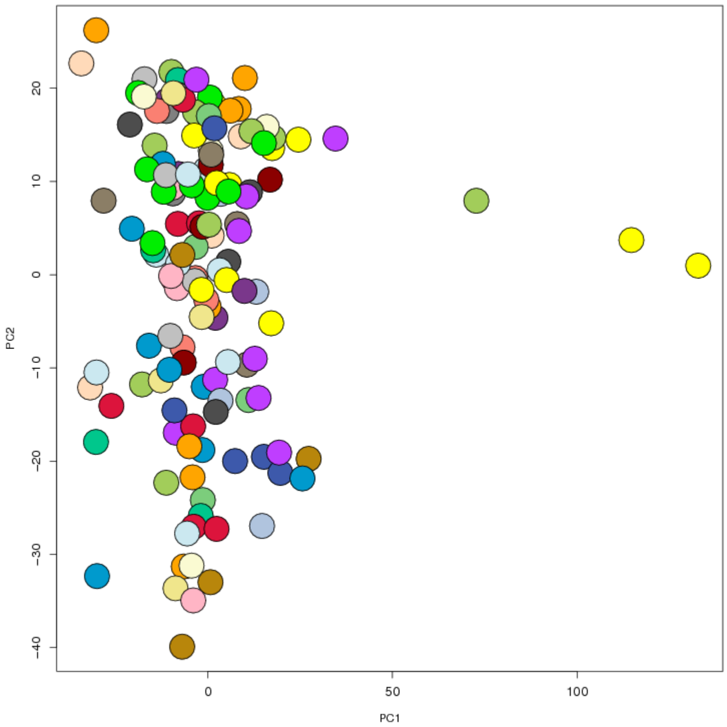

Principle Component Analysis

Principle Component Analysis (PCA) is a method commonly used to cluster data, i.e. to group data (samples) that are most similar. It is designed to capture the variance in a dataset in terms of principle components. In effect, one is trying to reduce the dimensionality of the data to summarise the most important (i.e. defining) parts whilst simultaneously filtering out noise. We would typically expect samples to cluster into treatment groups and/or sample source.

PCA can also be used to assess batch effects as samples processed on different days or with different reagents may cluster into separate groups. The PCA plot here has been conducted on RMA normalised data.

References

1. Gentleman R.C., Carey V.J., Bates D.M., et. al. Bioconductor: open software development for computational biology and bioinformatics Genome Biol. 2004; 5(10): R80.

2. Irizarry RA, Hobbs B, Collin F, et.al. Exploration, normalization, and summaries of high density oligonucleotide array probe level data.Biostatistics. 2003 Apr;4(2):249-64.

3. http://www.affymetrix.com/support/downloads/manuals/data_analysis_fundamentals_manual.pdf

---

If you would like to provide feedback on the format or content of this Quality Assessment report, please e-mail us at microarray@imperial.ac.uk
